# From the Gut to the Brain: Is Microbiota a New Paradigm in Parkinson’s Disease Treatment?

**DOI:** 10.3390/cells13090770

**Published:** 2024-04-30

**Authors:** Cristiana Vilela, Bruna Araújo, Carla Soares-Guedes, Rita Caridade-Silva, Joana Martins-Macedo, Catarina Teixeira, Eduardo D. Gomes, Cristina Prudêncio, Mónica Vieira, Fábio G. Teixeira

**Affiliations:** 1 Center for Translational Health and Medical Biotechnology Research (TBIO)/Health Research Network (RISE-Health), ESS, Polytechnic of Porto, R. Dr. António Bernardino de Almeida 400, 4200-072 Porto, Portugal; cristianasvilela@gmail.com (C.V.); cguedes@i3s.up.pt (C.S.-G.); edu04gomes@gmail.com (E.D.G.); cprudencio@ess.ipp.pt (C.P.); mav@ess.ipp.pt (M.V.); 2 Life and Health Sciences Research Institute (ICVS), School of Medicine, University of Minho, Campus de Gualtar, 4710-057 Braga, Portugal; bruna.araujo@i3s.up.pt (B.A.); joanamace@gmail.com (J.M.-M.); 3 ICVS/3B’s Associate Lab, PT Government Associated Lab, 4710-057/4805-017 Braga/Guimarães, Portugal; 4 i3S—Instituto de Investigação e Inovação em Saúde, Universidade do Porto, R. Alfredo Allen 208, 4200-135 Porto, Portugal; rcaridade@i3s.up.pt (R.C.-S.); cteixeira@i3s.up.pt (C.T.)

**Keywords:** Parkinson’s disease, microbiome, gut–brain axis

## Abstract

Parkinson’s disease (PD) is recognized as the second most prevalent primary chronic neurodegenerative disorder of the central nervous system. Clinically, PD is characterized as a movement disorder, exhibiting an incidence and mortality rate that is increasing faster than any other neurological condition. In recent years, there has been a growing interest concerning the role of the gut microbiota in the etiology and pathophysiology of PD. The establishment of a brain–gut microbiota axis is now real, with evidence denoting a bidirectional communication between the brain and the gut microbiota through metabolic, immune, neuronal, and endocrine mechanisms and pathways. Among these, the vagus nerve represents the most direct form of communication between the brain and the gut. Given the potential interactions between bacteria and drugs, it has been observed that the therapies for PD can have an impact on the composition of the microbiota. Therefore, in the scope of the present review, we will discuss the current understanding of gut microbiota on PD and whether this may be a new paradigm for treating this devastating disease.

## 1. Introduction

Parkinson’s disease (PD) is the second most common neurodegenerative disease of the central nervous system (CNS) [1,2]. Currently, this movement disorder affects 10 million people worldwide, with 75,000 new cases per year [3,4]. According to the World Health Organization (WHO), PD’s incidence and death rate is increasing faster than any other neurological condition, and its prevalence has doubled over the past 25 years [5].

PD is a complex and multifactorial disease, presenting distinctive pathological hallmarks, including the depletion of dopaminergic neurons in the substantia nigra pars compacta (SNpc). This depletion arises from the accumulation of a pathological misfolding alpha-synuclein (αSyn) protein, leading to the formation of Lewy neurites in the (remaining) neurons. αSyn is a monomeric protein and thus is expected to undergo genetic and post-translational modifications, including phosphorylation, ubiquitination, nitration, and oxidation, which can predispose it to misfold [2,6]. This misfolding makes this protein insoluble since it tends to form β-sheet-rich amyloid aggregates that accumulate and form intracellular eosinophilic inclusions [1,3]. Consequently, these changes can significantly impact the correct functioning of the central, peripheral, and enteric nervous system (ENS) [1,3,7,8]. In fact, the presence of αSyn aggregates, combined with other factors, such as mitochondrial dysfunction, ubiquitin-proteasome system (UPS), synaptic dysfunction, e.g., contributes to neuronal degeneration and death, leading to the appearance of motor (slow movements, resting tremors, rigidity, postural instability, and bradykinesia) and non-motor (dementia, gastrointestinal dysfunction, behavioral and cognitive impairments, among others) symptoms [1,6,7,9,10,11,12].

Despite all this knowledge, the etiology of PD is still unclear, although genetic and environmental factors provide the most plausible explanation for the onset of this pathology [7]. From the clinical point of view, PD displays heterogeneity, with different onset and progression patterns [13,14]. Advanced age at diagnosis is the most critical risk factor, although young Parkinsonism (even if rare) cannot be excluded. Nevertheless, in addition to aging, other risk factors should also be considered, namely family history of the disease, gender (in which men are more affected than women), and environmental factors such as exposure to pesticides and rural living [1,6]. Notwithstanding, it is known that cellular and molecular alterations can also contribute to PD through high levels of oxidative stress, mitochondrial dysfunction, neuroinflammation, xenobiotic toxins, and altered dopamine metabolism [6].

At the moment, both pharmacological and non-pharmacological therapies are available for PD. The current gold-standard PD treatment is pharmacological, based on dopamine replacement. However, non-pharmacological alternative approaches such as deep brain stimulation (DBS), an established safe neurosurgical symptomatic therapy, can be used in certain patients in more advanced stages of the disease, in which the treatment had no effectiveness or in which the medication itself produced severe side effects [15,16]. Nevertheless, these treatments promote temporary relief, being ineffective all over time, leading to the necessity of increasing drug dosages (in case of pharmacological therapies) or DBS intensities (in case of non-pharmacological approaches), which in most cases results in the appearance of adverse effects such as sleep disturbance, impulsivity, and addiction [1,4]. Hence, addressing this remains a noteworthy constraint in managing PD and a significant obstacle in developing or repurposing innovative therapies to slow down or halt its progression [16]. Moreover, it poses the question of how we can effectively manage PD and determine the optimal target for its treatment.

Despite numerous other avenues of research, in recent years, significant emphasis has been placed on exploring the vast potential of the gut microbiota and its intricate interconnection. As a result, efforts have been made to comprehend how the gut microbiome may respond under PD conditions, specifically whether it may contribute to the development or progression of the disease or offer a new therapeutic target to address PD. Therefore, in the present review, we will explore the bidirectional association between the gut microbiota and PD, discussing the current understanding of the gut microbiota in the pathophysiology of PD and as a potential target for novel therapeutic approaches.

## 2. Gut Microbiome

The human body is over 99% microbial in terms of genes. Microbiota is a complex ecological community composed of all the microorganisms that reside in our gut, and the term microbiome has been coined to refer to the entire genetic material (genome) of the microbiota [17]. Our gut holds almost 100 trillion microbes, meaning there are 10–100 times more bacteria than eukaryotic cells in the human body [10,18]. Nevertheless, bacterial composition and diversity differ according to different parts of the body [6]. The colonization of the gut is thought to begin immediately after birth, and it can be influenced by various factors such as the method of removing the fetus, namely vaginal or cesarean, or the type of breastfeeding (breastfeeding or formula), environmental stimuli, and antibiotic use [17,19,20]. Remarkably, this colonization is considered in the first 3–5 years of life. Although it is generally stable within healthy adult individuals throughout life, it is a dynamic entity whose composition is susceptible to change due to external disturbances [6,21]. Factors such as genetics, stress, environmental exposures, age, metabolism, geography, surgeries, physical activity, antibiotic intake, and diet can influence the stability of human gut microbiota [17,21].

Specifically, microbiota comprises bacteria, eukaryotic cells, viruses, archaeobacteria, fungi, and protozoa [22]. These microbiota constituents interact with each other and the host, impacting health through their metabolic activities and host interactions and in normal human physiology and disease contexts [6,18]. Bacteria predominantly control the gut microbiota’s metabolic activities, such as modulation of energy metabolism, nutrition absorption, and regulation of gut microbiota composition [23]. Thus, gut microbes can play a harmless or beneficial effect on the host, being essential to keep the gut homeostasis [4,24]. In addition, the microbiota interacts with the immune system, facilitating the normal development of immune functions during homeostasis and the emission of signals to promote the maturation of immune cells [24].

**Figure 1:**
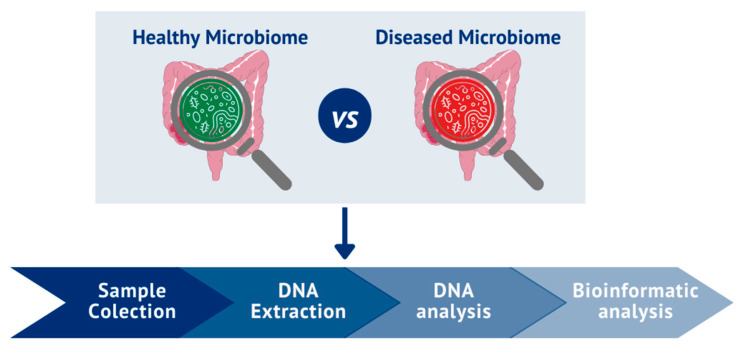
Phases of gut microbiome study.

The adult microbiota harbors more than 1000 phylotypes of bacteria at the species level, with Firmicutes and Bacteroides emerging as the two most abundant phyla, representing at least 70–75% of the total number of microorganisms [25]. Actinobacteria, Proteobacteria, and Verrucomicrobia can also be found, albeit in a smaller amount [21,26]. Scientific reports have been highlighting that it is essential to understand the stability of the microbiota within an individual over time to predict health status and to be able to develop therapies to correct dysbiosis, the term used to define any imbalance between the bacterial community and the host. Indeed, gut dysbiosis is associated with several pathologies, including systemic or neurological disorders [18,26]. Accordingly, recent studies have claimed that gut microbiome can be an important piece of evidence for understanding human pathology and physiology better [3]. Along with that, even though there was variability in adult gut communities, certain bacterial populations were common across different groups of individuals. Thus, a concept called “enterotype” emerged, which has been used to categorize individuals according to the composition of their intestinal microbiota [7]. Based on this concept, it was then proposed that three distinct symbiotic host-microbial states could be formed, driven by groups of dominant genera, namely Bacteroides (this has recently been subdivided into two groups: B1 and B2), Prevotella, or Ruminococcus. However, it should be noted that this concept of enterotype remains to be fully addressed [7,18,20,21]. Nevertheless, several factors may also play a role in this distinction and characterization. For instance, there is considerable intra- and inter-individual diversity in the microbiome of healthy control subjects and in the context of multiple disorders, where intestinal dysbiosis has been described, indicating that the gut microbiota is not uniform across populations. Environmental factors (e.g., smoking, alcohol, and diet), which significantly influence the structure and composition of microbial communities, can be used to explain this heterogeneity [7,27]. In addition, age, genetics, and lifestyle are factors that also affect microbiota (see Figure 1); these are also critical factors contributing to PD onset and modulation throughout life [26].

### 2.1. Age

Microbiota colonization begins immediately after birth and plays an essential role in developing the ENS and postnatal immune system [4,7]. This colonization process is primarily influenced by various perinatal conditions, such as the type of birth, nutrition, and the use or non-use of medication in the initial stages [28]. During the first years of life, the gut microbiota experiences various compositional and functional changes, which can significantly impact individual susceptibility to immune-related diseases in adulthood [7]. In the case of neurodegenerative diseases, aging is a well-defined hallmark associated with the accumulation of mitochondrial DNA defects, oxidative damage, and neuromelanin [26,29]. Furthermore, it is also recognized that problems caused by the gut or gut bacteria that eventually lead to dysbiosis have a higher frequency in older people [3,26].

### 2.2. Genetics

PD is mainly considered an idiopathic condition, with only approximately 10–15% of cases having a hereditary component associated with several genetic mutations [30]. Extensive research has been focusing on SNCA (αSyn), LRRK2 (leucine-rich repeat kinase-2), PINK-1 (phosphatase and tensin [PTEN] homologue-induced kinase-1), and PARK genes [31,32]. SNCA gene encodes for αSyn, and its mutations impact the protein’s expression levels, thus becoming a risk factor for PD. This gene has been widely associated with increased opportunistic pathogens in the intestine of PD patients. Interestingly, mutations in SNCA are not the prevalent ones in PD patients. Indeed, LRRK-2 is the most frequent genetic cause of the autosomal dominant form of PD, accounting for 10–40% of familial cases in various ethnicities [33]. Mutations in this enzyme are also closely associated with inflammatory bowel diseases (IBDs) [33,34].

The PINK-1 gene plays a role in the removal of damaged mitochondria; mutations in this gene are associated with mitochondrial dysfunction, as seen in the autosomal recessive early-onset PD [35]. The PARK gene encodes the Parkin protein, which can undergo several mutations, resulting in a cellular accumulation of damaged mitochondria [31]. In addition, mutations in this protein are also known to disrupt the ubiquitin-proteasome pathway, which ultimately triggers the accumulation of misfolded protein aggregates (e.g., αSyn) [31,36]. Curiously, exposure to pathogenic bacteria or bacterial metabolites was shown to cause epigenetic changes in the genes mentioned above. Hence, this interaction could be involved in most sporadic cases of PD. However, the relationship between genetic predisposition and the bacterial community in a PD context remains poorly understood [6].

### 2.3. Environment

Increased xenobiotic degradation in the gut, particularly herbicides and pesticides, has been found in the gut of PD patients [6,37]. Preclinical studies have demonstrated that exposure to these compounds can induce the death of dopaminergic neurons, leading to movement impairments in mice [6,38]. Still, considering the wide variety of pesticides in use, their long half-life, and the potential for accumulation in food chains, the associated risk may be even greater [39]. Therefore, more extensive studies should be conducted to approach this potential interconnection and how this may interplay with microbiome and neurologic diseases.

### 2.4. Lifestyle

**Figure 2:**
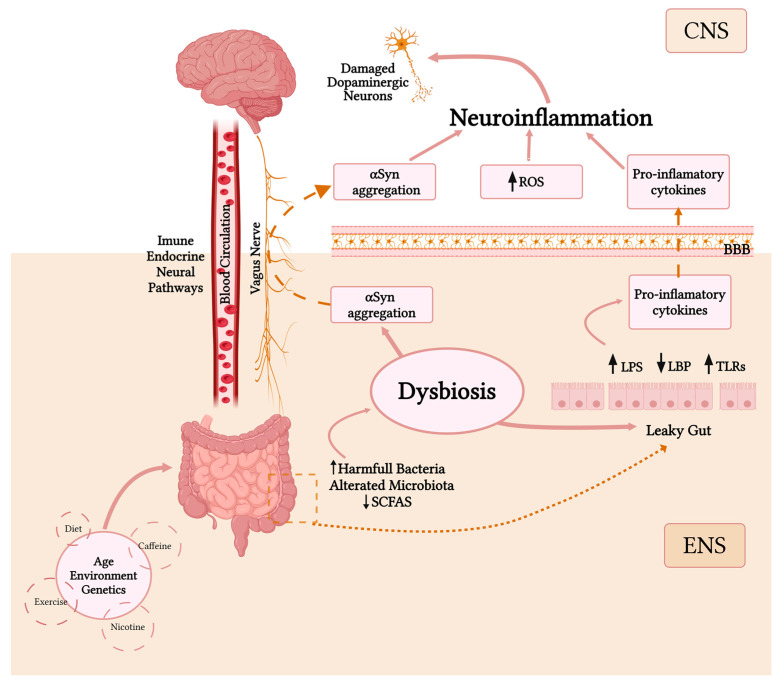
Gut–brain axis model. Age, environment, and genetics are the three major factors modulating the microbiota. In addition, there are other lifestyle factors that appear to play a role in PD pathophysiology. These factors will cause an alteration in the microbiota, increasing harmful bacteria and decreasing SCFAs, which will lead to a state of dysbiosis; this causes the integrity and function of the intestinal barrier to be affected, increasing metabolites such as LPS and TLRs and decreasing LBP, promoting an increase in various pro-inflammatory cytokines that in turn will cross the BBB reaching the CNS. This state of dysbiosis will also cause an aggregation of αSyn, which will reach the CNS via the vagus nerve (Braak’s Theory). In the brain, this aggregation of αSyn increases pro-inflammatory cytokines, and increased ROS will lead to a state of neuroinflammation and consequently lead to the death of dopaminergic neurons. Parkinson's Disease (PD); short-chain fatty acids (SCFAs); bacterial lipopolysaccharide (LPS); lipopolysaccharide-binding protein (LBP); Toll-like receptors (TLRs); blood–brain barrier (BBB); enteric nervous system (ENS); central nervous system (CNS); alpha-synuclein (α-Syn); reactive oxygen species (ROS). The figure was generated using BioRender.com (accessed in 1 March 2024).

Although the three factors mentioned above (age, genetics, and environment) have major effects on the pathophysiology of PD, lifestyle can also play a role in PD, in which diet, physical exercise, caffeine, and nicotine consumption could be contributors (Figure 2) [39]. Exercise has been associated with beneficial alterations in microbiome composition, impacting energy homeostasis and regulation [4,40]. In addition to medication, it is clinically recommended to prescribe activation therapies, such as physiotherapy, which is a widely employed therapeutic approach [39]. Caffeine consumption is one of the most studied nutritional habits and is highly correlated with a decreased PD risk, suggesting its potential to modulate the pathophysiology of the disease [41,42,43,44]. As regards nicotine, there are a few studies that have consistently demonstrated an inverse association between smoking and the risk of developing PD [41,42]. Former smokers and smokers had a lower risk of developing PD compared to non-smokers [44]. Despite all the information regarding possible microbiota modulators and their impact on such disorders, a fundamental question remains: how can microbiome data analysis be correlated with a diseased state?

The main challenge in addressing this question lies in several perspectives, namely in the heterogeneity profile between patients and diseases and in the technological availability to deeply discriminate the inherent variances. The conjugation between advanced techniques (proteomics, metabolomics, transcriptomics) and bioinformatic tools will be crucial to analyzing and integrating large amounts of data, based on the idea that only then will it be possible to capture the multilayered structure of the data.

## 3. Microbiome Characterization Techniques

The study of human microbiota diversity began in the 1680s when Anton Van Leeuwenhoek compared the oral and fecal microbiotas and found that microorganisms vary not only from region to region but also between states of health or disease regardless of location [45,46,47]. The initial investigations into the microbiota, namely the identification and characterization of microorganisms, were conducted through methodologies focused on cultivating and isolating bacteria [48]. Nevertheless, it quickly became evident that all existing species’ cultivation and phenotypic characterization were unfeasible, either due to a lack of favorable conditions or to slow bacterial growth [49]. Nevertheless, developing novel analytical and sequencing techniques for studying the human microbiota has become a priority [47]. Indeed, the study of the microbiota has been a significant focus of various diseases in recent years, with an increasing number of areas recognizing the microbiota as a “Key Player” in several pathological conditions [50,51,52]. Presently, a multitude of molecular biological analysis tools can be employed in the study of the gut microbiota, from conventional to more advanced techniques, as outlined in Table 1 [53,54,55]. Although the development of traditional methods has been essential to the initial investigations of the gut microbiota, the development of novel methodologies, such as microbiome shotgun sequencing, has made it possible to overcome the biases associated with traditional culture, thus enabling a better understanding of the composition, diversity, and interrelationships among the microorganisms constituting the microbiota, contributing significantly to our understanding of their role in states of health or disease [55].

The microbiota is studied by analyzing the structure and dynamics of bacterial communities and the interactions that they can establish with each other [75]. Regardless of the chosen analytical technique, the study comprises four essential phases (Figure 1): (1) fecal sample collection, (2) DNA extraction, (3) DNA analysis (utilizing the most suitable technique for the intended purpose (techniques outlined in Table 1)), and (4) bioinformatic analysis of the results obtained. These analyses can be conducted to obtain taxonomy profiling or an analysis of the gene functions of the gut microbiome [53,76]. Subsequently, correlations between the healthy and altered gut microbiome in a pathological context can be made [53,77,78].

Bearing this in mind, to perform microbiome research accurately, it is essential that, in combination with standard methodologies, appropriate pipelines (such as Quantitative Insights Into Microbial Ecology (QIIME) [79], and the Metagenomic Rapid Annotation using Subsystem Technology (MG-RAST) [80] should be established for research regarding the human gut microbiome [48,53,81]. This is crucial since methodological differences could lead to inconsistent data, thus limiting knowledge in this area [53,82,83]. Projections indicate that, in the forthcoming years, analyses of the gut microbiome could yield pivotal insights, facilitating the provision of personalized medicine for different diseases and thereby exerting a profound impact on individual human health [84,85,86].

Despite the substantial growth in this area in recent years, it is still impossible to have direct correlations between the microorganisms in our gut microbiota and their role in the onset and progression of specific pathologies, such as PD [55,87]. Consequently, there is a paramount need to allocate resources towards comprehending intestinal microorganisms’ diversity and their bioactive metabolites’ functionality in the context of diverse diseases, including PD [88,89]. This strategic investment is essential for the eventual utilization of the intestinal microbiome in diagnosing and treating PD.

## 4. Microbiome and PD: From Diagnosis to Treatment

Exposure to pathogenic bacteria, or bacterial metabolites, is one of the factors shown to cause epigenetic changes when they interact with (PD) genes [6]. It has been proposed that this interaction may be involved in most sporadic cases of PD. However, the relationship between genetic predisposition and the gut bacterial community in PD needs to be better understood [6]. These features lead to a particular interest in exploring the gut microbiome when studying PD. From a scientific and clinical perspective, this holds a vast potential for diagnosis, prognosis, and even for understanding disease pathogenesis, as dysbiosis can induce peripheral inflammation, which, in turn, may contribute to the pathophysiology of PD through humoral and neural pathways [33,90]. Numerous studies have revealed a correlation between the prevalence of specific bacterial taxa and the longevity of the disease, severity, motor and non-motor symptom scores, and the use of antiparkinsonian therapies [6]. Furthermore, a recent analysis showed that 42.29% of underweight PD cases are mediated by the gut microbiome through a decrease in SCFA-producing bacteria and an increase in proinflammatory bacteria, indicating that the gut microbiome may have some impact on the PD mechanism [91]. Additionally, thirteen studies conducted across three continents have demonstrated differences in the gut bacterial community between PD patients and healthy individuals [92].

This bidirectional communication between the brain and the gut microbiota is known as the brain–gut microbiome axis (GBrA), and in the last years, there has been an exponential growth in research around the axis, particularly in the applications that the GBrA could have in PD [93]. The GBrA refers to the interaction between the gut microbes and the CNS via metabolic, immune, neuronal, and endocrine signaling pathways and mechanisms (Figure 2) [94]. Still, one pathway is offering a more direct form of communication: the vagus nerve [3,4,6,41,94]. The vagus nerve is a complex bidirectional system that allows communication between the hypothalamic-pituitary-adrenal axis hormones, the CNS, the autonomic nervous system, and the ENS [4,26]. Modifications in this axis have been postulated to impact the development of PD directly [3,4]. The discovery of αSyn aggregates in peripheral locations, such as the ENS, supports the concept of the “GBrA”. The ENS, which innervates the gastrointestinal (GI) system and is located near the intestinal lumen, provides a significant region for interaction with bacteria [6,7]. From the gut to the brain, there is an ascending aggregation of αSyn and Lewy Bodies (LB) formation, leading to progressive neurodegeneration [4]. Having mentioned this, a study conducted by Sampson et al. underscores the significance of GBrA in the pathogenesis of PD [95]. The research involving transgenic mice with PD reveals that alterations in the intestinal composition contribute to the disease’s pathogenesis [95]. Notably, αSyn aggregates demonstrate a propensity to readily spread from the enteric nervous system to the brain, shedding light on the pivotal role of the GBrA in the progression of PD [4,95].

In this way, it can be assumed that the intestinal microbiota profoundly influences this entire neuronal network, facilitating the absorption of nutrients, vitamins, and medications while modulating the immune system [4]. In 2017, Stolzenberg et al. demonstrated a positive correlation between inflammation of the intestinal wall in pediatric patients and the expression of αSyn in the ENS [96]. This finding suggests an inflammatory response that precedes GI infections commonly associated with the pathogenesis of PD [97]. Additionally, it further indicates that the expression of this protein aggregates within the ENS is part of its normal immune defense mechanism, which supports the hypothesis of Braak et al. (See below) [6,96].

## 5. Microbiome vs. αSyn—Potential Contributions of the Microbiome to PD: Braak’s Hypothesis

Interestingly, PD not only affects the brain, but recent studies have also demonstrated that the disease extends to other organs, namely belonging to the GI system [33]. In 2003, Braak and colleagues postulated a “dual-hit hypothesis” suggesting that αSyn aggregation, triggered by a neurotrophic agent or an unknown pathogen, propagates in a prion-like manner from the ENS and the olfactory bulb to the CNS via the dorsal motor nucleus of the vagus nerve (DMV) [4,26,41,42,98]. The DMV, together with the nucleus tractus solitarius and the nucleus ambiguus, constitute the brainstem dorsal vagal complex, which acts as a central control of visceral functions, such as gastrointestinal function, including gastric motility [99,100]. The hypothesis that this retrograde transport happens through the DMV and not the other two structures that make up the brainstem dorsal vagal complex is due to the fact that the DMV (unlike the nucleus tractus solitarius and the nucleus ambiguus) is an unmyelinated structure, which makes it suitable for axonal transport [101]. This invasion creates a pro-inflammatory environment, increasing the permeability of the intestinal barrier and leading to reactive oxygen species (ROS) accumulation. Consequently, this will result in dysregulation in homeostasis, activating various immunological mechanisms that might promote αSyn aggregation [33,102]. Based on such postulation, Braak et al. proposed a six-stage progression system for PD in the brain and in surrounding olfactory regions based on the propagation patterns of αSyn, which can be further correlated with several characteristic hallmarks of the disease [33,103]. With this in mind, Holmqvist et al. conducted studies that provided direct evidence supporting Braak’s hypothesis [8]. This study observed that all forms of αSyn (monomers, oligomers, and fibrils) were actively transported from the gut to the brain through the DMV. To support this theory, the same authors employed human brain lysate from PD patients containing various forms of αSyn and recombinant αSyn in an *in vivo* model (Adult wild-type Sprague Dawley rats) [8]. In both situations, it was deduced that αSyn was transported from the gut to the brain via the vagus nerve [8]. Following such pieces of evidence, S. Kim et al., upon injection of misfolded αSyn into the gut of healthy mice, discovered an accumulation of this protein in both the vagus nerve and the brain. This finding points to the fact that PD may originate in the gut in specific subsets of patients [41,104]. Yet, this assumption remains under debate.

Given the divergent nature of the symptoms associated with PD, these findings support the hypothesis that this disease may be divided into two variants. One variant postulates that PD originates in the gut, explaining why some patients initially present intestinal discomfort problems. The other variant suggests that the disease initiates in the brain, being justified by the early onset of neurological symptoms [41,105].

## 6. Gut Microbiota and Neuroinflammation

The cell structure of microbes varies, presenting a tendency to initiate signaling pathways for pattern recognition receptors that can cause inflammation [4]. Inflammation is widely recognized as a prominent pathophysiological characteristic of PD [9]. Patients with PD have been observed to display heightened levels of several inflammatory molecules (IL-1β (Interleukin-1 beta), IL-6 (Interleukin-6), INF-γ (interferon-γ), MCP-1 (Monocyte chemoattractant protein-1) and TNF-α (Tumor necrosis factor-α)) in their bloodstream, cerebrospinal fluid (CSF), and brain [106]. (Neuro)Inflammation is widely recognized as a prominent pathophysiological characteristic of PD, being a major contributor to disease progression and severity, so it can be assumed that it typically develops due to protein aggregation and dopaminergic cell death [9,26,92]. Indeed, several studies have demonstrated the ability of αSyn to stimulate the production of pro-inflammatory cytokines in both microglia and monocytes [6]. Nevertheless, it should be noted that the same inflammatory markers are not always implicated in PD. Even so, some specific cytokines and chemokines are frequently dysregulated (in the brain, CSF and blood) such as IL-6, TNF (Tumor necrosis factor), IL-1 (Interleukin-1), CRP (C-reactive protein), IL-10 (Interleukin 10), CCL5 (Chemokine (C-C motif) ligand 5), INF-γ, and IL-2 (Interleukin-2) [4,26]. This inflammatory process may be triggered by a breakdown in intestinal barrier function (caused, for example, by bacterial infections, oxidative stress, and dysbiosis), leading to increased systemic exposure to inflammatory microbial products, causing αSyn deposition, a characteristic of intestinal hyper-permeability in PD (Figure 2) [4,6,92,107]. This may be related to the etiology and symptomatology of PD [42]. Furthermore, systemic inflammation can disrupt the blood–brain barrier (BBB) permeability, resulting in inflammatory cytokines or systemic immune cells’ entrance into the CNS. As a result, the association between low-grade inflammation in the gut and PD has gained considerable attention [26,92].

The GI tract is coated on the inside by the intestinal mucosa, which is a physical and immunological barrier that separates the external environment from the host’s bloodstream. Numerous factors can compromise this barrier’s proper functioning and permeability, including bacteria and their metabolic by-products. For instance, disruption of the bacterial balance causes an increase in permeability, subsequently triggering an inflammatory intestinal response, which leads to a state of (neuro)inflammation [6]. Indeed, comparative studies have shown that individuals with PD exhibit an overabundance of a pro-inflammatory profile (such as *Streptococcus*, *Bifidobacterium*, *Lactobacillus*, *Akkermansia*, and *Desulfovibrio*) in their gut microbiota, along with increased expression of genes related to pro-inflammatory cytokines and chemokines in their intestinal tissue, compared to control subjects [26,92,108]. In addition, a significant population of neurons near the intestinal epithelium directly connects to the brainstem via the vagus nerve [26]. In a study conducted by Sampson et al. in 2016, oral administration of microbial metabolites into germ-free mice promotes neuroinflammation (αSyn-Dependent Microglia Activation) and subsequently leads to the manifestation of characteristic motor symptoms of PD [33,95]. Nevertheless, the role of inflammation is still a matter of discussion in PD since studies defend that it can exert a dual role, being either neurotoxic or neuroprotective [9].

## 7. Molecular Mediators: Toll-like Receptors (TLR)

The vagus nerve is frequently involved in how the microbiota influences the CNS, specifically through neuroimmune and neuroendocrine mechanisms [94,109]. This communication is facilitated by microbe-derived molecules known as modulators that interact with enteroendocrine cells (EECs), enterochromaffin cells (ECCs), and the mucosal immune system to propagate signals [94,110]. TLRs are innate immune receptors expressed in microglia, immune and non-immune cells [111]. They are capable of recognizing various exogenous and endogenous stimuli to mediate inflammatory responses [111,112]. Most studies indicated an increased expression of TLRs in the brain and peripheral blood cells of PD samples (TLR2, TLR4, and TLR9 having the most prominent roles) [111,112]. We can hypothesize that by increasing both TLRs and inflammatory cytokines (whose function is to serve as endogenous damage-associated molecular patterns (DAMPs)), αSyn may directly impact microglial cells [94,113]. The signaling of TLRs triggers NF-κB activation, which is essential for an increase in nitric oxide production and dopaminergic neuron apoptosis [6,114]. In fact, according to some researchers, when gut dysbiosis occurs, the barrier becomes dysfunctional, activating TLRs, which subsequently recognize bacterial lipopolysaccharide (LPS) [10]. The indirect effects of LPS on brain inflammation through vagal mediation have been established for a long time. Systemic exposure to LPS is responsible for a sickness behavior induction [115,116]. The presence of LPS impairs the function of the intestinal barrier and promotes the production of various pro-inflammatory cytokines that will cross the BBB, thereby inducing a state of neuroinflammation and consequently leading to neuronal cell death in PD [6,10].

## 8. Molecular Mediators: LPS and Lipopolysaccharide-Binding Protein (LBP)

Maintaining the integrity of the intestinal barrier is essential for regulating how luminal substances travel across the gut wall, both through paracellular and transcellular pathways. As previously mentioned, a compromised intestinal barrier increases the susceptibility of systemic circulation of inflammatory microbial products such as LPS [26,92]. This leads to intestinal inflammation and oxidative stress, further disrupting mucosal permeability and triggering the aggregation of αSyn in the ENS [4]. Actually, LPS is the major component of the outer surface membrane specific to gram-negative bacteria [117]. Studies regarding the blood and plasma of PD patients have shown increased systemic exposure to LPS, supporting the hypothesis that PD is associated with intestinal barrier dysfunction [92]. Given that oscillations between LPS and LBP levels occur, there have been indications assuming this is a potential marker for dysbiosis [26]. For instance, a study by Paul M. Carvey et al. showed that prenatal exposure to LPS leads to long-term loss of dopaminergic neurons in SNpc of a PD rat model [118]. Such a finding is in line with the study conducted by Adam Jangula and colleagues, which revealed that the expression of αSyn enhances LPS-induced BBB permeability in preclinical models of PD (Snca^−/−^ (KO) mice) [119].

## 9. Metabolic Mediators: Short-Chain Fatty Acids (SCFAs)

With the help of EECs and ECCs, SCFAs play a crucial role as neuroactive molecules in the communication between host and microbes [6,94]. The concentration and distribution of SCFA types significantly influence gut health and homeostatic functions, such as mucin production, tight-junction formation, glucose homeostasis, and immunomodulation [4,120]. From a physiological perspective, these compounds are produced in the gut through bacterial metabolism of carbohydrates and protein, serving as an energy source for microbial growth and proliferation [6,92]. The three main types of SCFAs are acetic, propionic, and butyric acid [92]. Nevertheless, in environments with high pH levels, SCFAs may exist as salts, which limits their penetration through the bacterial cell wall [94].

One of the primary mediators contributing to the impact of the gut on PD symptoms is the reduced concentrations of a specific SCFA, butyrate [42]. Such decreased levels of these SCFAs in the intestine severely affect the integrity and function of the intestinal barrier, promoting inflammation and increasing the risk of αSyn deposition [6]. Thus, when a host has a diet low in fermentable fiber, the microbes nourish themselves on mucus glycans using alternative energy sources, which are, in turn, less favorable. As a result, fermentative activity decreases, reducing SCFA production [94]. Butyrate exerts its effects through multiple mechanisms, including activating specific receptors and inhibiting the histone deacetylase (HDAC) enzyme [121]. By inhibiting HDAC, butyrate can regulate gene expression and epigenetic activity, influencing various cellular processes (such as proliferation and apoptosis) [121]. Moreover, butyrate enhances mitochondrial performance and prevents cell death by blocking a key mediator (mitochondrial complex Ι) [42,122]. Additionally, it is thought that the microbiota may influence the synthesis of molecules such as neurotransmitters (e.g., gamma amino butyric acid) and the products of fermentation (e.g., the short-chain fatty acids butyrate, propionate, and acetate) promoting the functioning of the nervous system [6,123]. While contrasting fecal SCFA levels in PD patients and control subjects, decreased concentrations of SCFAs, particularly butyric acid, were found in PD patients, which were linked to microbiota alterations [92]. In fact, several bacterial taxa reportedly reduced in PD are SCFA producers [2,3,26,92]. These molecules are important as an energy source in the body, as well as by stimulating colonic blood flow, capturing fluids and electrolytes, influencing inflammatory responses, and proliferating the mucosa [92,94].

Thus, through the actions of SCFAs on tight junction protein expression, they can modulate the permeability of both intestinal and blood–brain barriers, exhibiting anti-inflammatory and antioxidant properties [6,26].

## 10. Microbiome: A New Source for PD Biomarkers?

As previously indicated, in addition to the fact that there is no effective treatment to halt the progression of the disease, when patients are diagnosed, they already have a high level of degeneration of the dopaminergic neurons [78,124]. Consequently, there is an urgent need to develop sensitive and practical biomarkers that might allow the detection of PD at earlier stages, particularly within the symptomatic prodromic phases of the disease [125,126,127].

Numerous molecules in the CSF, including αSyn, DJ-1, amyloid-beta, tau, and lysosomal enzymes, are currently under investigation as potential biomarkers for PD [7,128,129,130]. Recently, Siderowf et al. have shown that α-Syn seed amplification assays (SAAs) might be able to distinguish PD patients and healthy subject profiles, which can be tested in the near future as a potential biomarker [131]. To date, no definitive and specific biomarkers have been identified. Nevertheless, an in-depth analysis of gut dysbiosis might be an important step to successfully originate specific PD biomarkers.

Current research indicates that the intestinal microbiota and the GI environment exert influence and may be used as non-motor biomarkers for PD [95]. As previously noted, alongside the characteristic motor symptoms, individuals afflicted with PD commonly experience GI dysfunctions, primarily involving the intestinal tract [132,133]. Constipation, a common GI disorder in PD, may be partly caused by changes in gut microbiota composition and its byproducts [134,135]. Consequently, constipation, dysbiosis, modified intestinal permeability, enteric accumulation of αSyn, and GI inflammation are evident in individuals indicative of PD at various points in their lives [136,137,138]. It is hypothesized that by profiling the microbiota of PD patients and those at a heightened risk of developing PD, alterations in the GI system could be employed as potential biomarkers for the early detection of PD [139]. However, more studies are needed to support this possibility fully.

The genomic characterization of fecal samples offers a potential avenue for identifying biomarkers associated with PD [140,141]. Qian and colleagues successfully identified twenty-five genetic markers within the intestinal microbiota, which is beneficial for PD diagnosis and to distinguish this pathology from others, such as Alzheimer’s disease (AD) and multisystem atrophy (MSA) [141]. Furthermore, investigations into the fecal metabolome of individuals with PD, in comparison to healthy controls, revealed a diminished concentration of SCFAs and noteworthy alterations in the metabolism of amino acids, lipids, and vitamins [142,143]. Therefore, bacterial metabolites and perturbed metabolic pathways may also serve as biomarkers for PD [143]. In addition, a recent study showed differences between PD patients and controls. This study showed three metabolites that differed in dietary habits (carbohydrates, trans fatty acids, and potassium) between PD patients and controls. In addition to this change, there was also a change in the relative abundance of 6 bacterial genes [144]. Despite these promising findings, to date, no studies have specifically evaluated the fecal metabolome as a diagnostic biomarker for PD. While the obtained developments are encouraging, replication in larger population samples is imperative to ascertain their reproducibility. Additionally, an emphasis on characterizing the intestinal microbiota, not only through gene sequencing but also at the level of the fecal metabolome, is essential to uncover novel possibilities for biomarkers associated with PD. Additionally, exploring biomarkers, underlying causes of the disease, and symptom management strategies offer potential clinical applications in the future. Pursuing these avenues holds the potential not only to enhance our understanding of PD but also to contribute to the development of effective and early interventions aimed at symptom management, slowing down the disease progression in the future and making it possible to identify the patients who would most benefit from experimental therapy and to quantify the effectiveness of future drugs [145].

## 11. Microbiota: Are We Looking for a New Therapeutic Target?

The existing therapies for PD, including the gold standard, levodopa, predominantly alleviate symptoms with limited efficacy and lack substantial prophylactic effects [136,146,147]. Given the prevalence of GI dysfunction in over 80% of PD individuals, it is plausible to hypothesize that alterations in our microbiota may influence PD. Therefore, this suggests the potential role of a compromised GBrA in the pathogenesis of the disease [148,149].

In contrast to predictive biomarkers, which might indicate or demonstrate a causal relationship, therapeutic interventions need a direct connection between microbes and disease states [150]. If imbalances in the microbiota were considered synonymous with disease, restoring a healthy microbiota emerges as a compelling therapeutic alternative [151]. In this context, diverse approaches have been investigated to model the microbiota and reinstate equilibrium. Indeed, microbiome-based therapies encompass various strategies, including live biotherapeutics, fecal microbiota transplant, microbiome mimetics, dietary interventions, prebiotics, probiotics, symbiotics, antibiotics, and phage therapy [150,152,153]. A recent double-blind, placebo-controlled, randomized, phase 2 trial suggests that fecal microbiota transplant may have a beneficial effect on motor symptoms in PD patients at an early stage of the disease, highlighting the potential of microbiota modulation in the treatment of PD [154].

Furthermore, since the intestinal microbiota is significantly influenced by diet, dietary interventions also emerge as a potential therapeutic target for preventing, modifying, or delaying PD [155,156,157,158]. Notably, nutritional interventions (such as probiotics) can restore the composition of the intestinal microbiota, thereby improving the prognosis of the disease [136,159].

As mentioned above, patients with this disease often exhibit dysregulated gut microbiota characterized by reduced SCFA bacteria and increased LPS bacteria [155,160,161]. Addressing this imbalance, a treatment method could involve using prebiotics, such as fibers, which can stimulate the growth of SCFA-producing bacteria, increasing SCFAs in the colon [162,163]. In addition, it was noted that administering prebiotics to individuals diagnosed with PD may potentially modify the course of the disease. Therefore, assumptions have been made postulating that an increase in SCFA levels might mitigate issues such as intestinal permeability to endotoxin, colonic inflammation, and neuroinflammation, ultimately contributing to a reduction in the loss of dopaminergic neurons [7,24,160].

Furthermore, Perez-Pardo et al. discovered that a dietary intervention combining two membrane synthesis precursors—uridine and docosahexaenoic acid (DHA)—which enhance phospholipid synthesis, along with prebiotic fibers, exhibited a neuroprotective effect in a PD model-induced by rotenone [164]. Their study demonstrated partial alleviation of both motor and non-motor symptoms induced by rotenone and the restoration of DAT levels in the striatum [164,165,166]. This suggests that the combination of these nutritional elements may benefit the progression and symptomatology of PD. Subsequent investigations have further corroborated this view, demonstrating that diet, probiotics, and prebiotics can potentially normalize dysbiotic microbiota [7,167,168,169]. Therefore, these interventions could hold promise as potentially beneficial strategies in treating PD.

Another potential use of microbiota-targeted intervention is in treating PD patients to optimize the efficacy of current PD drugs since a better understanding of the influence of gut microbiota as a predictive biomarker for drug metabolism will give us information on the bioavailability of the medication, its responsiveness, and the direct results of the treatment [4,170,171,172]. Notably, research demonstrated that eradicating *Helicobacter pylori (H. pylori)* improved levodopa absorption, a key drug used in PD treatment, resulting in reduced motor symptoms [173,174,175]. In PD patients, an *H. pylori* infection correlates with ongoing motor deterioration [174,175]. Therefore, *H. pylori* infection interferes with the pharmacokinetics of levodopa, leading to increased delays and periods of ineffectiveness [176]. These findings provide a compelling rationale for considering microbiota-targeted interventions to optimize levodopa therapy, particularly for individuals requiring high and frequent doses of levodopa who may experience significant side effects [177]. Such interventions could potentially enhance the therapeutic outcomes and alleviate the life-altering impact of levodopa-related side effects in PD patients.

While the prospect of interventions targeting the microbiota in PD is promising, it is crucial to acknowledge that additional clinical trials are necessary to ascertain the actual benefits in this context. Presently, there are 39 clinical trials underway (including those exploring the relationship between PD and gut dysbiosis and potential therapeutics), as outlined in Table 2 (Search conducted on the National Library of Medicine’s Clinical Trials Registry Platform with the terms “Parkinson’s” and “Gut Microbiome”). However, it is essential to note that pre-clinical and clinical studies investigating gut therapeutic strategies for diverse PD symptoms come with certain limitations. Importantly, small sample sizes, insufficient duration for therapeutic intervention, variations in disease severity among patients, inadequacies in outcome measures, and a lack of standardization in key reading variables are some of those limitations [136,178]. Furthermore, the substantial heterogeneity in the microbiome across individuals raises concerns regarding gut microbiota variability in PD patients [179,180]. This emphasizes the significance of individualized approaches during clinical assessment, diagnosis, and treatment selection in pursuing more effective outcomes [181].

## 12. Gut Microbiota–Drug Interaction for the Treatment of PD

Notably, the efficacy of pharmaceutical drugs exhibits considerable variability among individuals, owing to the intricate nature of the human body [182,183]. The complexity of this system is further underscored by the pivotal role played by the gut microbiota [184]. Recent research has illuminated the significant influence exerted by gut bacteria on the modulation of drug mechanisms and associated side effects [185,186]. Importantly, these interactions between gut microbes and pharmaceutical agents are bidirectional [187]. In other words, drugs can induce alterations in the composition and functionality of the gut microbiota, subsequently impacting the metabolism and utilization of these drugs within the body [188,189].

The spectrum of pharmaceuticals demonstrating discernible connections to the microbiota is extensive. However, the specific alterations incurred by various drugs on the microbiota exhibit distinctive patterns. Consequences may encompass a diminution in microbial diversity and an augmentation in the proliferation of particular potentially pathogenic bacteria, such as *Proteobacteria*, *Bifidobacteriaceae*, *Ruminococcaceae*, *Verrucomicrobiaceae*, and *Christensenellaceae* [190,191,192,193,194]. Nevertheless, the mechanisms through which medications impact the microbiota predominantly operate through indirect mechanisms, wherein alterations are introduced to various facets of the gut environment, including nutrient composition, thereby exerting an influence on bacterial growth [195]. Notably, certain medications undergo direct metabolic transformations by bacteria, thereby dictating their subsequent distribution and functionality within the body [186]. An illustrative instance of this phenomenon is evident in the case of levodopa. In this context, bacteria directly metabolize levodopa, ultimately shaping its trajectory and effects within the organism.

Following oral administration, the absorption of levodopa is imperative through the small intestine for it to traverse the BBB and gain access to the brain [196,197]. Within the brain, the human enzyme aromatic amino acid decarboxylase (AAAD), also called DOPA decarboxylase, catalyzes the conversion of levodopa into therapeutically active dopamine [198,199,200]. The pivotal determinant of the drug’s efficacy lies in the bioavailability of levodopa to the brain [201]. Consequently, levodopa is frequently co-administered with catechol metabolism inhibitors, exemplified by carbidopa, to impede its metabolism at sites other than its intended action, thereby optimizing its availability for therapeutic impact [198,202]. This is crucial, as certain gut bacteria and peripheral human enzymes can metabolize the drug before it crosses the BBB [110,203]. This preemptive metabolism could substantially diminish the availability and consequently undermine the efficacy of levodopa [203]. In light of this, the interplay between levodopa and *H. pylori*, as previously mentioned, underscores the intricate relationship between pharmaceutical agents and gut microbiota [176].

Recent research has brought another dimension of microbial involvement in levodopa metabolism, specifically through microbial decarboxylases in the gut [204]. Notably, a newly identified bacterial metabolism of levodopa, facilitated by tyrosine decarboxylases (tyrDCs), has been determined, with *Enterococcus faecalis* (*E. faecalis*) predominantly carrying out this process [171]. Intriguingly, the potential to enhance the drug’s efficacy is evident through manipulating this microbial metabolism. Mutation of tyrDCs in *E. faecalis* has been shown to impede the bacterial conversion of Levodopa into dopamine, presenting a promising avenue for improving the drug’s therapeutic effectiveness [171,205].

Nevertheless, considering the role of the intestinal microbiota in drug metabolism, coupled with the substantial impact of medications on the microbiota’s composition, the possible interaction between PD medication and the microbiota’s composition is not surprising. Actually, insights derived from these studies support the plausible existence of a clinically significant relationship between the microbiota and drug metabolism in individuals with PD. Consequently, mapping the human microbiome becomes imperative to unravel the underlying mechanisms governing these intricate microbiota–drug interactions [206]. Such exploration is pivotal not only for elucidating the dynamics of this interaction but also for unveiling how it influences the efficacy of medication in the context of PD.

## 13. Future Perspectives

**Figure 3:**
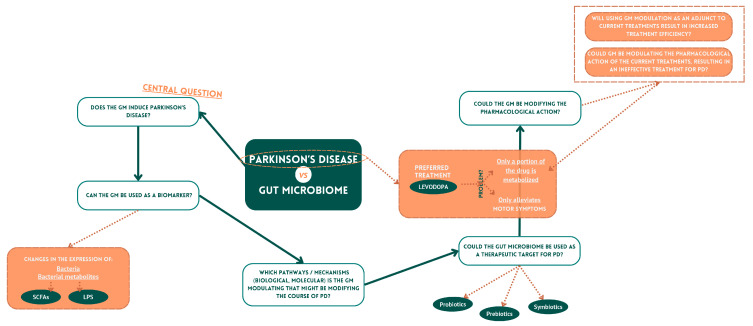
Gut microbiome and Parkinson’s disease. The gut microbiome is an incredibly complex ecosystem, having a denoted and multifaceted impact on people’s health. Indeed, it is nowadays well accepted that it might play a role in the pathogenesis of numerous diseases, including neurodegenerative diseases such as PD. This appreciation of the importance and complexity of the gut microbiome leads to several questions that are currently important to address, with the correct application of technological assays to the same set of samples in hopes of capturing multiple layers of information about the microbiome’s involvement in disease. Parkinson’s Disease (PD); gut microbiome (GM); short-chain fatty acids (SCFAs); bacterial lipopolysaccharide (LPS).

Although high-throughput sequencing technology has allowed outstanding advances in gut microbiota research, much remains to be unraveled. There is plenty of indirect evidence to support the active role of the microbiota in PD, but there is limited conclusive support. And the real question remains unanswered: Is gut microbiome dysregulation a cause or a consequence of PD? It is tough to demonstrate the exact molecular and cellular pathways through which the microbiota may promote the pathogenesis of PD. This is because the microbiota is highly heterogeneous between individuals. Most of the existing studies on gut microbes in PD are cross-sectional studies, which cannot sufficiently indicate the causal relationships between gut microbes and PD pathogenesis, resulting in a significant gap in the literature and much divergence in the results. Therefore, considering the high heterogeneity of gut microbes between different individuals, it is necessary to emphasize more longitudinal research to advance our knowledge about the mechanisms underlying the correlation between gut microbes and PD so that in the future, we can use gut microbiome as a biomarker and so that more targeted treatment strategies based on the gut microbiome can be developed (either alone or as an adjuvant for existing therapies) (Figure 3).

## 14. Conclusions

PD is a highly heterogeneous disease with an unclear etiology. Whether or not the microbiota is the initial cause of PD remains uncertain, but it has been hypothesized to play a key role in the distinct stages of the disease. This is supported by various pro-inflammatory mediators associated with bacteria, which might contribute to or facilitate a neuroinflammatory state in PD. The GBrA assumes particular importance in PD, namely in the formation of αSyn aggregates and in the bidirectional communication and transport of αSyn via the vagus nerve. Therefore, even though it is an exciting perspective, there are still questions that should be addressed: is microbiota alteration a cause or a consequence of PD? Can the microbiome be a potential source of biomarkers or a therapeutic target for PD? Can the microbiome be a critical modulator of the current/available treatment pipeline?

Considering this, future randomized controlled trials are needed to evaluate the potential impact of microbiota in the different stages of PD and distinct treatment regimens. By doing so, new concepts in the pathophysiology and therapeutic setting of the disease might be established.

## Figures and Tables

**Table 1:** Techniques used to characterize the gut microbiota.

Technique	Description	References
Culture-based	Isolation/Growth of bacteria on selective media	[54,55,56,57]
16S rRNA (16S ribosomal RNA) gene sequencing based on cloning	The process involves cloning the entire 16S rRNA amplicon, followed by Sanger sequencing and capillary electrophoresis for analysis.	[54,55,58,59]
Direct sequencing of 16S rRNA amplicons	High-throughput parallel sequencing of partial 16S rRNA amplicons.	[54,55,60]
Quantitative real-time polymerase chain reaction (qPCR)	16S rRNA amplification and quantification. A substance in the reaction mixture exhibits fluorescence when it is attached to a double-stranded DNA.	[54,55,61,62]
Denaturing gradient gel electrophoresis (DGGE)/Temperature Gradient Gel Electrophoresis (TGGE)	16S rRNA amplicons separated on a gel using a denaturant/temperature gradient	[54,55,63]
Terminal restriction fragment length polymorphism (T-RFLP)	After the quantification of fluorescently labeled primers, the 16S rRNA amplicon undergoes digestion utilizing restriction enzymes. Subsequently, the resulting digested fragments are separated through gel electrophoresis.	[54,55,64]
Fluorescence in situ hybridization (FISH)	Oligonucleotide probes, labeled with fluorescent markers, bind to sequences that are complementary to the target 16S rRNA. The fluorescence generated during this hybridization process can be quantified using flow cytometry.	[54,55,65]
Microbiome Shotgun sequencing	Extensive parallel sequencing of the entire genome.	[54,55]
Metagenomics	Exploring high-resolution profiling of gut microbiota genomes and characterizing gene structures of uncultivated microbiota.	[54,66]
Metatranscriptomics	High-resolution gene expression profiling is achieved through sequencing messenger RNA (mRNA) or complementary DNA (cDNA). This approach is used to explore differential microbial gene expression under various physiological or environmental conditions, providing insights into microbial adaptation and responses.	[67,68]
Metaproteomics	High-resolution protein monitoring and profiling involve the identification of proteins and peptides, enabling the examination of differential microbial protein production in diverse physiological or environmental conditions.	[69,70,71]
Metabolomics	Metabolites undergo analysis to profile the metabolic activity of microbial hosts.	[70,72,73]
DNA microarrays	Oligonucleotide probes labeled with fluorescent markers undergo hybridization with complementary nucleotide sequences. The resulting fluorescence is detected using a fluorescence laser detector.	[54,74]

**Table 2:** Summary table of clinical cases registered (Clinical Trials.gov: accessed on 4 February 2024).

Clinical Trial Title	Study Type	Sample Size	Study Phase	ClinicalTrials.gov Identifier
Levodopa Response and Gut Microbiome in Patients With Parkinson’s Disease	Observational	38	Completed	NCT04956939
Prebiotics in the Parkinson’s Disease Microbiome	Interventional	20	Completed	NCT04512599
Microbiota Modification for the Treatment of Motor Complication of Parkinson’s Disease	Interventional	14	Completed	NCT04730245
A Trial of Fecal Microbiome Transplantation in Parkinson’s Disease Patients	Interventional	51	Completed	NCT04854291
Determining the Microbiota Composition of the Middle Meatus in Parkinson’s	Observational	48	Completed	NCT03336697
Study of the Fecal Microbiome in Patients With Parkinson’s Disease	Interventional	15	Completed	NCT03671785
Increased Gut Permeability to Lipopolysaccharides (LPS) in Parkinson’s Disease	Observational	43	Completed	NCT01155492
Fecal Microbiota Transplantation for Parkinson’s Disease	Interventional	49	Completed	NCT03808389
Gut Microbiota Across Early Stages of Synucleinopathy: From High-risk Relatives, REM Sleep Behavior Disorder to Early Parkinson’s Disease	Observational	441	Completed	NCT03645226
Mediterranean Diet Intervention to Improve Gastrointestinal Function in Parkinson’s Disease: a Randomized, Controlled, Clinical Trial (MEDI-PD)	Interventional	46	Completed	NCT04683900
Fecal Microbiota Transplantation As a Potential Treatment for Parkinson’s Disease	Interventional	10	Completed	NCT03876327
Metabolic Cofactor Supplementation in Alzheimer’s Disease (AD) and Parkinson’s Disease (PD) Patients	Interventional	120	Completed	NCT04044131
Constipation and Changes in the Gut Flora in Parkinson’s Disease	Observational	80	Recruiting	NCT05787756
AADC/TDC in Advanced Parkinson’s Disease	Observational	50	Recruiting	NCT05558787
Parkinson’s Disease Biomarkers in Nerve Cells in the Gut	Observational	60	Recruiting	NCT05347407
Efficacy and Safety of Fecal Microbiota Transplantation in the Treatment of Parkinson’s Disease With Constipation	Interventional	30	Recruiting	NCT04837313
Microbiota Intervention to Change the Response of Parkinson’s Disease	Interventional	86	Recruiting	NCT03575195
Microbiome Composition and Function Contributes to Cognitive Impairment and Neuroinflammation in Parkinson’s Disease	Observational	100	Recruiting	NCT05419453
Gut Microbiota in the Progression of Alpha-synucleinopathies	Observational	490	Recruiting	NCT05353868
Meridian Activation Remedy System for Parkinson’s Disease (MARS-PD)	Interventional	88	Recruiting	NCT05621772
The Sunnybrook Dementia Study (SDS)	Observational	1600	Recruiting	NCT01800214
N-DOSE: A Dose Optimization Trial of Nicotinamide Riboside in Parkinson’s Disease	Interventional	80	Recruiting	NCT05589766
Resistant Maltodextrin for Gut Microbiome in Parkinson’s Disease: Safety and Tolerability Study	Interventional	30	Active, not recruiting	NCT03667404
Gut Health and Probiotics in Parkinson’s (SymPD)	Interventional	60	Active, not recruiting	NCT05146921
MOVIN’ CARE for PD (Risk Management) (jcpdmcP)	Interventional	308	Active, not recruiting	NCT06147284
MOVIN’ CARE for PD (Mind-body Interventions) (jcpdmcI)	Interventional	154	Active, not recruiting	NCT06078046
Ketogenic Diet Interventions in Parkinson’s Disease: Safeguarding the Gut Microbiome (KIM)	Interventional	50	Not yet recruiting	NCT05469997
Microbiome and Diet in Parkinson’s Disease—a Randomized, Controlled Phase 2 Trial (PD-Diet)	Interventional	40	Not yet recruiting	NCT06207136
Observational Small Intestine and Blood Fingerprint (SmIle) Study in Parkinson’s Disease	Observational	100	Not yet recruiting	NCT06003608
Efficacy of Probiotics for Parkinson Disease (PD)	Interventional	300	Not yet recruiting	NCT06118294
Effects of Probiotics on Peripheral Immunity in Parkinson’s Disease	Interventional	88	Enrolling by invitation	NCT05173701
Establishment of a Human Tissue Bank for Studying the Microbial Etiology of Neurodegenerative Diseases	Observational	0	Withdrawn	NCT01954875
Characterization of Fecal Microbiome Changes After Administration of PRIM-DJ2727 in Parkinson’s Disease Patients	Interventional	0	Withdrawn	NCT03026231
Single-center Pathophysiological Study of the Role of Inflammation, Changes in the Intestinal Epithelial Barrier and the Intestinal Microbiota in Parkinson’s Disease	Interventional	77	Terminated	NCT04652843
Study of the Genome, Gut Metagenome and Diet of Patients With Incident Parkinson’s Disease	Observational	138	Terminated	NCT04119596
Gut Microbiota and Parkinson’s Disease	Observational	50	Unknown status	NCT03710668
The Microbiome in Parkinson’s Disease	Observational	210	Unknown status	NCT03129451
Deep Brain Stimulation Therapy and Intestinal Microbiota	Observational	30	Unknown status	NCT04855344
A Pilot Study to Explore the Role of Gut Flora in Parkinson’s Disease	Observational	100	Unknown status	NCT04148326

## Data Availability

Not applicable.

## References

[1] Balestrino R., Schapira A.H.V. (2020). *Parkinson Disease*. Eur. J. Neurol..

[2] Parnetti L., Gaetani L., Eusebi P., Paciotti S., Hansson O., El-Agnaf O., Mollenhauer B., Blennow K., Calabresi P. (2019). CSF and Blood Biomarkers for Parkinson’s Disease. Lancet. Neurol..

[3] Zhu X., Li B., Lou P., Dai T., Chen Y., Zhuge A., Yuan Y., Li L. (2021). The Relationship between the Gut Microbiome and Neurodegenerative Diseases. Neurosci. Bull..

[4] Lubomski M., Tan A.H., Lim S.-Y., Holmes A.J., Davis R.L., Sue C.M. (2020). Parkinson’s Disease and the Gastrointestinal Microbiome. J. Neurol..

[5] World Health Organization (2023). Parkinson Disease.

[6] Pavan S., Prabhu A.N., Prasad Gorthi S., Das B., Mutreja A., Shetty V., Ramamurthy T., Ballal M. (2022). Exploring the Multifactorial Aspects of Gut Microbiome in Parkinson’s Disease. Folia Microbiol. (Praha).

[7] Keshavarzian A., Engen P., Bonvegna S., Cilia R. (2020). The Gut Microbiome in Parkinson’s Disease: A Culprit or a Bystander?. Prog. Brain Res..

[8] Holmqvist S., Chutna O., Bousset L., Aldrin-Kirk P., Li W., Björklund T., Wang Z.-Y., Roybon L., Melki R., Li J.-Y. (2014). Direct Evidence of Parkinson Pathology Spread from the Gastrointestinal Tract to the Brain in Rats. Acta Neuropathol..

[9] Hassanzadeh K., Rahimmi A. (2018). Oxidative Stress and Neuroinflammation in the Story of Parkinson’s Disease: Could Targeting These Pathways Write a Good Ending?. J. Cell. Physiol..

[10] Sun M.F., Shen Y.Q. (2018). Dysbiosis of Gut Microbiota and Microbial Metabolites in Parkinson’s Disease. Ageing Res. Rev..

[11] Lim K.L., Tan J.M.M. (2007). Role of the Ubiquitin Proteasome System in Parkinson’s Disease. BMC Biochem..

[12] Gómez-Benito M., Granado N., García-Sanz P., Michel A., Dumoulin M., Moratalla R. (2020). Modeling Parkinson’s Disease With the Alpha-Synuclein Protein. Front. Pharmacol..

[13] Wüllner U., Borghammer P., Choe C., Csoti I., Falkenburger B., Gasser T., Lingor P., Riederer P. (2023). The Heterogeneity of Parkinson’s Disease. J. Neural Transm..

[14] Greenland J.C., Williams-Gray C.H., Barker R.A. (2019). The Clinical Heterogeneity of Parkinson’s Disease and Its Therapeutic Implications. Eur. J. Neurosci..

[15] Hariz M., Blomstedt P. (2022). Deep Brain Stimulation for Parkinson’s Disease. J. Intern. Med..

[16] Virel A., Dudka I., Laterveer R., Af Bjerkén S. (2019). (1)H NMR Profiling of the 6-OHDA Parkinsonian Rat Brain Reveals Metabolic Alterations and Signs of Recovery after N-Acetylcysteine Treatment. Mol. Cell. Neurosci..

[17] Cryan J.F., O’Riordan K.J., Sandhu K., Peterson V., Dinan T.G. (2020). The Gut Microbiome in Neurological Disorders. Lancet. Neurol..

[18] Clemente J.C., Ursell L.K., Parfrey L.W., Knight R. (2012). The Impact of the Gut Microbiota on Human Health: An Integrative View. Cell.

[19] Diaz Heijtz R., Wang S., Anuar F., Qian Y., Björkholm B., Samuelsson A., Hibberd M.L., Forssberg H., Pettersson S. (2011). Normal Gut Microbiota Modulates Brain Development and Behavior. Proc. Natl. Acad. Sci. USA.

[20] Wang Y., Kasper L.H. (2014). The Role of Microbiome in Central Nervous System Disorders. Brain. Behav. Immun..

[21] Foster J.A., McVey Neufeld K.-A. (2013). Gut-Brain Axis: How the Microbiome Influences Anxiety and Depression. Trends Neurosci..

[22] Cox M.J., Cookson W.O.C.M., Moffatt M.F. (2013). Sequencing the Human Microbiome in Health and Disease. Hum. Mol. Genet..

[23] Liu J., Tan Y., Cheng H., Zhang D., Feng W., Peng C. (2022). Functions of Gut Microbiota Metabolites, Current Status and Future Perspectives. Aging Dis..

[24] Zhu M., Liu X., Ye Y., Yan X., Cheng Y., Zhao L., Chen F., Ling Z. (2022). Gut Microbiota: A Novel Therapeutic Target for Parkinson’s Disease. Front. Immunol..

[25] Bull M.J., Plummer N.T. (2014). Part 1: The Human Gut Microbiome in Health and Disease. Integr. Med. (Encinitas).

[26] Shannon K.M. (2022). Gut-Derived Sterile Inflammation and Parkinson’s Disease. Front. Neurol..

[27] Ahn J., Hayes R.B. (2021). Environmental Influences on the Human Microbiome and Implications for Noncommunicable Disease. Annu. Rev. Public Health.

[28] Milani C., Duranti S., Bottacini F., Casey E., Turroni F., Mahony J., Belzer C., Delgado Palacio S., Arboleya Montes S., Mancabelli L. (2017). The First Microbial Colonizers of the Human Gut: Composition, Activities, and Health Implications of the Infant Gut Microbiota. Microbiol. Mol. Biol. Rev..

[29] Reeve A., Simcox E., Turnbull D. (2014). Ageing and Parkinson ’ s Disease: Why Is Advancing Age the Biggest Risk Factor?. Ageing Res. Rev..

[30] Deng H., Wang P., Jankovic J. (2018). The Genetics of Parkinson Disease. Ageing Res. Rev..

[31] Biswas S., Bagchi A. (2022). Study of the Effects of Nicotine and Caffeine for the Treatment of Parkinson’s Disease. Appl. Biochem. Biotechnol..

[32] Nuytemans K., Theuns J., Cruts M., Van Broeckhoven C. (2010). Genetic Etiology of Parkinson Disease Associated with Mutations in the SNCA, PARK2, PINK1, PARK7, and LRRK2 Genes: A Mutation Update. Hum. Mutat..

[33] Chao Y.-X., Gulam M.Y., Chia N.S.J., Feng L., Rotzschke O., Tan E.-K. (2020). Gut-Brain Axis: Potential Factors Involved in the Pathogenesis of Parkinson’s Disease. Front. Neurol..

[34] Liu Z., Lenardo M.J. (2012). The Role of LRRK2 in Inflammatory Bowel Disease. Cell Res..

[35] Vizziello M., Borellini L., Franco G., Ardolino G. (2021). Disruption of Mitochondrial Homeostasis: The Role of Pink1 in Parkinson’s Disease. Cells.

[36] Hernán M.A., Takkouche B., Caamaño-Isorna F., Gestal-Otero J.J. (2002). A Meta-Analysis of Coffee Drinking, Cigarette Smoking, and the Risk of Parkinson’s Disease. Ann. Neurol..

[37] Hill-Burns E.M., Debelius J.W., Morton J.T., Wissemann W.T., Lewis M.R., Wallen Z.D., Peddada S.D., Factor S.A., Molho E., Zabetian C.P. (2017). Parkinson’s Disease and Parkinson’s Disease Medications Have Distinct Signatures of the Gut Microbiome. Mov. Disord..

[38] Blesa J., Phani S., Jackson-Lewis V., Przedborski S. (2012). Classic and New Animal Models of Parkinson’s Disease. J. Biomed. Biotechnol..

[39] Reichmann H., Csoti I., Koschel J., Lorenzl S., Schrader C., Winkler J., Wüllner U. (2022). Life Style and Parkinson’s Disease. J. Neural Transm..

[40] Gaßner H., Steib S., Klamroth S., Pasluosta C.F., Adler W., Eskofier B.M., Pfeifer K., Winkler J., Klucken J. (2019). Perturbation Treadmill Training Improves Clinical Characteristics of Gait and Balance in Parkinson’s Disease. J. Parkinsons. Dis..

[41] Considine E., Yin L., Hartmann M. (2021). A Review of the Primary Nutritional and Environmental Factors Associated with Parkinson’s Disease. J. Student Res..

[42] Bjørklund G., Dadar M., Anderson G., Chirumbolo S., Maes M. (2020). Preventive Treatments to Slow Substantia Nigra Damage and Parkinson’s Disease Progression: A Critical Perspective Review. Pharmacol. Res..

[43] Postuma R.B., Lang A.E., Munhoz R.P., Charland K., Pelletier A., Moscovich M., Filla L., Zanatta D., Romenets S.R., Altman R. (2012). Caffeine for Treatment of Parkinson Disease: A Randomized Controlled Trial. Neurology.

[44] Gallo V., Vineis P., Cancellieri M., Chiodini P., Barker R.A., Brayne C., Pearce N., Vermeulen R., Panico S., Bueno-De-Mesquita B. (2019). Exploring Causality of the Association between Smoking and Parkinson’s Disease. Int. J. Epidemiol..

[45] Leevven A., Obfervatioiis M. (1684). An Abstract of a Letter from Mr. Anthony Leevvenhoeck at Delft, Dated Sep. 17. 1683. Containing Some Microscopical Observations, about Animals in the Scurf of the Teeth, the Substance Call’d Worms in the Nose, the Cuticula Consisting of Scales. R. Soc..

[46] Power A., Dobell C. (1920). The Discovery of the Intestinal Protozoa of Man. Proc. R. Soc. Med..

[47] Ursell L.K., Metcalf J.L., Parfrey L.W., Knight R. (2012). Defining the Human Microbiome. Nutr. Rev..

[48] Grice E.A., Segre J.A. (2012). Published in Final Edited Form as: The Human Microbiome: Our Second Genome. Annu. Rev. Genom. Hum. Genet..

[49] Hollister E.B., Gao C., Versalovic J. (2014). Compositional and Functional Features of the Gastrointestinal Microbiome and Their Effects on Human Health. Gastroenterology.

[50] Blum H.E. (2017). The Microbiome: A Key Player in Human Health and Disease. J. Healthc. Commun..

[51] Hou K., Wu Z.X., Chen X.Y., Wang J.Q., Zhang D., Xiao C., Zhu D., Koya J.B., Wei L., Li J. (2022). Microbiota in Health and Diseases. Signal Transduct. Target. Ther..

[52] Afzaal M., Saeed F., Shah Y.A., Hussain M., Rabail R., Socol C.T., Hassoun A., Pateiro M., Lorenzo J.M., Rusu A.V. (2022). Human Gut Microbiota in Health and Disease: Unveiling the Relationship. Front. Microbiol..

[53] Song E.J., Lee E.S., Nam Y.D. (2018). Progress of Analytical Tools and Techniques for Human Gut Microbiome Research. J. Microbiol..

[54] Fraher M.H., O’Toole P.W., Quigley E.M.M. (2012). Techniques Used to Characterize the Gut Microbiota: A Guide for the Clinician. Nat. Rev. Gastroenterol. Hepatol..

[55] Rezasoltani S., Ahmadi Bashirzadeh D., Nazemalhosseini Mojarad E., Asadzadeh Aghdaei H., Norouzinia M., Shahrokh S. (2020). Signature of Gut Microbiome by Conventional and Advanced Analysis Techniques: Advantages and Disadvantages. Middle East J. Dig. Dis..

[56] Cani P.D. (2013). Gutmicrobiota and Obesity: Lessons from the Microbiome. Brief. Funct. Genom..

[57] Scales B.S., Huffnagle G.B. (2013). The Microbiome in Wound Repair and Tissue Fibrosis. J. Pathol..

[58] Suau A., Bonnet R., Sutren M., Godon J.J., Gibson G.R., Collins M.D., Doré J. (1999). Direct Analysis of Genes Encoding 16S RRNA from Complex Communities Reveals Many Novel Molecular Species within the Human Gut. Appl. Environ. Microbiol..

[59] Hayashi H., Sakamoto M., Benno Y. (2002). Phylogenetic Analysis of the Human Gut Microbiota Using 16S RDNA Clone Libraries and Strictly Anaerobic Culture-Based Methods. Microbiol. Immunol..

[60] Matsuo Y., Komiya S., Yasumizu Y., Yasuoka Y., Mizushima K., Takagi T., Kryukov K., Fukuda A., Morimoto Y., Naito Y. (2021). Full-Length 16S RRNA Gene Amplicon Analysis of Human Gut Microbiota Using MinION^TM^ Nanopore Sequencing Confers Species-Level Resolution. BMC Microbiol..

[61] Shukla R., Ghoshal U., Dhole T.N., Ghoshal U.C. (2015). Fecal Microbiota in Patients with Irritable Bowel Syndrome Compared with Healthy Controls Using Real-Time Polymerase Chain Reaction: An Evidence of Dysbiosis. Dig. Dis. Sci..

[62] Furet J.P., Firmesse O., Gourmelon M., Bridonneau C., Tap J., Mondot S., Doré J., Corthier G. (2009). Comparative Assessment of Human and Farm Animal Faecal Microbiota Using Real-Time Quantitative PCR. FEMS Microbiol. Ecol..

[63] Siqueira J.F., Rôças I.N., Rosado A.S. (2005). Application of Denaturing Gradient Gel Electrophoresis (DGGE) to the Analysis of Endodontic Infections. J. Endod..

[64] Imaeda H., Fujimoto T., Takahashi K., Kasumi E., Fujiyama Y., Andoh A. (2012). Terminal-Restriction Fragment Length Polymorphism (T-RFLP) Analysis for Changes in the Gut Microbiota Profiles of Indomethacin-and Rebamipide-Treated Mice. Digestion.

[65] Namsolleck P., Thiel R., Lawson P., Holmstrøm K., Rajilic M., Vaughan E.E., Rigottier-Gois L., Collins M.D., De Vos W.M., Blaut M. (2004). Molecular Methods for the Analysis of Gut Microbiota. Microb. Ecol. Health Dis..

[66] Wang W.L., Xu S.Y., Ren Z.G., Tao L., Jiang J.W., Zheng S.S. (2015). Application of Metagenomics in the Human Gut Microbiome. World J. Gastroenterol..

[67] Bashiardes S., Zilberman-Schapira G., Elinav E. (2016). Use of Metatranscriptomics in Microbiome Research. Bioinform. Biol. Insights.

[68] Ojala T., Kankuri E., Kankainen M. (2023). Understanding Human Health through Metatranscriptomics. Trends Mol. Med..

[69] Issa Isaac N., Philippe D., Nicholas A., Raoult D., Eric C. (2019). Metaproteomics of the Human Gut Microbiota: Challenges and Contributions to Other OMICS. Clin. Mass Spectrom..

[70] Peters D.L., Wang W., Zhang X., Ning Z., Mayne J., Figeys D. (2019). Metaproteomic and Metabolomic Approaches for Characterizing the Gut Microbiome. Proteomics.

[71] Wang Y., Zhou Y., Xiao X., Zheng J., Zhou H. (2020). Metaproteomics: A Strategy to Study the Taxonomy and Functionality of the Gut Microbiota. J. Proteomics.

[72] Smirnov K.S., Maier T.V., Walker A., Heinzmann S.S., Forcisi S., Martinez I., Walter J., Schmitt-Kopplin P. (2016). Challenges of Metabolomics in Human Gut Microbiota Research. Int. J. Med. Microbiol..

[73] Vernocchi P., Del Chierico F., Putignani L. (2016). Gut Microbiota Profiling: Metabolomics Based Approach to Unravel Compounds Affecting Human Health. Front. Microbiol..

[74] Wang R.F., Beggs M.L., Erickson B.D., Cerniglia C.E. (2004). DNA Microarray Analysis of Predominant Human Intestinal Bacteria in Fecal Samples. Mol. Cell. Probes.

[75] Bikel S., Valdez-Lara A., Cornejo-Granados F., Rico K., Canizales-Quinteros S., Soberón X., Del Pozo-Yauner L., Ochoa-Leyva A. (2015). Combining Metagenomics, Metatranscriptomics and Viromics to Explore Novel Microbial Interactions: Towards a Systems-Level Understanding of Human Microbiome. Comput. Struct. Biotechnol. J..

[76] Nearing J.T., Comeau A.M., Langille M.G.I. (2021). Identifying Biases and Their Potential Solutions in Human Microbiome Studies. Microbiome.

[77] Madhogaria B., Bhowmik P., Kundu A. (2022). Correlation between Human Gut Microbiome and Diseases. Infect. Med..

[78] Vijay A., Valdes A.M. (2022). Role of the Gut Microbiome in Chronic Diseases: A Narrative Review. Eur. J. Clin. Nutr..

[79] Caporaso J.G., Kuczynski J., Stombaugh J., Bittinger K., Bushman F.D., Costello E.K., Fierer N., Peña A.G., Goodrich J.K., Gordon J.I. (2010). QIIME Allows Analysis of High-Throughput Community Sequencing Data. Nat. Methods.

[80] Meyer F., Paarmann D., D’Souza M., Olson R., Glass E.M., Kubal M., Paczian T., Rodriguez A., Stevens R., Wilke A. (2008). The Metagenomics RAST Server—A Public Resource for the Automatic Phylogenetic and Functional Analysis of Metagenomes. BMC Bioinform..

[81] Morgan X.C., Huttenhower C. (2014). Meta’omic Analytic Techniques for Studying the Intestinal Microbiome. Gastroenterology.

[82] Lyu R., Qu Y., Divaris K., Wu D. (2023). Methodological Considerations in Longitudinal Analyses of Microbiome Data: A Comprehensive Review. Genes.

[83] Custer G.F., Gans M., van Diepen L.T.A., Dini-Andreote F., Buerkle C.A. (2023). Comparative Analysis of Core Microbiome Assignments: Implications for Ecological Synthesis. mSystems.

[84] Mukherjee S., Joardar N., Sengupta S., Sinha Babu S.P. (2018). Gut Microbes as Future Therapeutics in Treating Inflammatory and Infectious Diseases: Lessons from Recent Findings. J. Nutr. Biochem..

[85] Behrouzi A., Nafari A.H., Siadat S.D. (2019). The Significance of Microbiome in Personalized Medicine. Clin. Transl. Med..

[86] Kashyap P.C., Chia N., Nelson H., Segal E., Elinav E. (2017). Microbiome at the Frontier of Personalized Medicine. Mayo Clin. Proc..

[87] Ji B., Nielsen J. (2015). From Next-Generation Sequencing to Systematic Modeling of the Gut Microbiome. Front. Genet..

[88] Benameur T., Porro C., Twfieg M.E., Benameur N., Panaro M.A., Filannino F.M., Hasan A. (2023). Emerging Paradigms in Inflammatory Disease Management: Exploring Bioactive Compounds and the Gut Microbiota. Brain Sci..

[89] Krishnan S., Alden N., Lee K. (2015). Pathways and Functions of Gut Microbiota Metabolism Impacting Host Physiology. Curr. Opin. Biotechnol..

[90] Jia X., Chen Q., Zhang Y. (2023). Multidirectional Associations between the Gut Microbiota and Parkinson’s Disease, Updated Information from the Perspectives of Humoral Pathway, Cellular Immune Pathway and Neuronal Pathway. Front. Cell. Infect. Microbiol..

[91] Shih L.C., Lin R.J., Chen Y.L., Fu S.C. (2024). Unravelling the Mechanisms of Underweight in Parkinson’s Disease by Investigating into the Role of Gut Microbiome. npj Park. Dis..

[92] Aho V.T.E., Houser M.C., Pereira P.A.B., Chang J., Rudi K., Paulin L., Hertzberg V., Auvinen P., Tansey M.G., Scheperjans F. (2021). Relationships of Gut Microbiota, Short-Chain Fatty Acids, Inflammation, and the Gut Barrier in Parkinson’s Disease. Mol. Neurodegener..

[93] Chen L., Chen J., Wu M., Yan P., Zhou X. (2024). Analyzing the Bibliometrics of Brain-Gut Axis and Parkinson’s Disease. Front. Neurol..

[94] Martin C.R., Osadchiy V., Kalani A., Mayer E.A. (2018). The Brain-Gut-Microbiome Axis. Cell. Mol. Gastroenterol. Hepatol..

[95] Sampson T.R., Debelius J.W., Thron T., Janssen S., Shastri G.G., Ilhan Z.E., Challis C., Schretter C.E., Rocha S., Gradinaru V. (2016). Gut Microbiota Regulate Motor Deficits and Neuroinflammation in a Model of Parkinson’s Disease. Cell.

[96] Stolzenberg E., Berry D., Yang D., Lee E.Y., Kroemer A., Kaufman S., Wong G.C.L., Oppenheim J.J., Sen S., Fishbein T. (2017). A Role for Neuronal Alpha-Synuclein in Gastrointestinal Immunity. J. Innate Immun..

[97] Chen Q.Q., Haikal C., Li W., Li J.Y. (2019). Gut Inflammation in Association With Pathogenesis of Parkinson’s Disease. Front. Mol. Neurosci..

[98] Visanji N.P., Brooks P.L., Hazrati L.N., Lang A.E. (2014). The Prion Hypothesis in Parkinson’s Disease: Braak to the Future. Acta Neuropathol. Commun..

[99] Browning K.N., Travagli R.A. (2019). Central Control of Gastrointestinal Motility. Curr. Opin. Endocrinol. Diabetes Obes..

[100] Clyburn C., Browning K.N. (2021). Glutamatergic Plasticity within Neurocircuits of the Dorsal Vagal Complex and the Regulation of Gastric Functions. Am. J. Physiol.-Gastrointest. Liver Physiol..

[101] Braak H., Rüb U., Gai W.P., Del Tredici K. (2003). Idiopathic Parkinson’s Disease: Possible Routes by Which Vulnerable Neuronal Types May Be Subject to Neuroinvasion by an Unknown Pathogen. J. Neural Transm..

[102] Rietdijk C.D., Perez-Pardo P., Garssen J., van Wezel R.J.A., Kraneveld A.D. (2017). Exploring Braak’s Hypothesis of Parkinson’s Disease. Front. Neurol..

[103] Hawkes C.H., Del Tredici K., Braak H. (2007). Parkinson’s Disease: A Dual-Hit Hypothesis. Neuropathol. Appl. Neurobiol..

[104] Kim S., Kwon S.H., Kam T.I., Panicker N., Karuppagounder S.S., Lee S., Lee J.H., Kim W.R., Kook M., Foss C.A. (2019). Transneuronal Propagation of Pathologic α-Synuclein from the Gut to the Brain Models Parkinson’s Disease. Neuron.

[105] Borghammer P., Van Den Berge N. (2019). Brain-First versus Gut-First Parkinson’s Disease: A Hypothesis. J. Parkinsons. Dis..

[106] Zimmermann M., Brockmann K. (2022). Blood and Cerebrospinal Fluid Biomarkers of Inflammation in Parkinson’s Disease. J. Parkinsons. Dis..

[107] Di Vincenzo F., Del Gaudio A., Petito V., Lopetuso L.R., Scaldaferri F. (2023). Gut Microbiota, Intestinal Permeability, and Systemic Inflammation: A Narrative Review. Intern. Emerg. Med..

[108] Nie S., Wang J., Deng Y., Ye Z., Ge Y. (2022). Inflammatory Microbes and Genes as Potential Biomarkers of Parkinson’s Disease. npj Biofilms Microbiomes.

[109] Wang S.Z., Yu Y.J., Adeli K. (2020). Role of Gut Microbiota in Neuroendocrine Regulation of Carbohydrate and Lipid Metabolism via the Microbiota-Gut-Brain-Liver Axis. Microorganisms.

[110] Dicks L.M.T. (2022). Gut Bacteria and Neurotransmitters. Microorganisms.

[111] Heidari A., Yazdanpanah N., Rezaei N. (2022). The Role of Toll-like Receptors and Neuroinflammation in Parkinson’s Disease. J. Neuroinflamm..

[112] Zhang Z., Liu Z., Lv A., Fan C. (2023). How Toll-like Receptors Influence Parkinson’s Disease in the Microbiome–Gut–Brain Axis. Front. Immunol..

[113] Isik S., Yeman Kiyak B., Akbayir R., Seyhali R., Arpaci T. (2023). Microglia Mediated Neuroinflammation in Parkinson’s Disease. Cells.

[114] Tansey M.G., Wallings R.L., Houser M.C., Herrick M.K., Keating C.E., Joers V. (2022). Inflammation and Immune Dysfunction in Parkinson Disease. Nat. Rev. Immunol..

[115] Dantzer R., O’Connor J.C., Freund G.G., Johnson R.W., Kelley K.W. (2008). From Inflammation to Sickness and Depression: When the Immune System Subjugates the Brain. Nat. Rev. Neurosci..

[116] Biesmans S., Meert T.F., Bouwknecht J.A., Acton P.D., Davoodi N., De Haes P., Kuijlaars J., Langlois X., Matthews L.J.R., Ver Donck L. (2013). Systemic Immune Activation Leads to Neuroinflammation and Sickness Behavior in Mice. Mediators Inflamm..

[117] Alexander C., Rietschel E.T. (2001). Bacterial Lipopolysaccharides and Innate Immunity. J. Endotoxin Res..

[118] Carvey P.M., Chang Q., Lipton J.W., Ling Z. (2003). Prenatal Exposure to the Bacteriotoxin Lipopolysaccharide Leads to Long-Term Losses of Dopamine Neurons in Offspring: A Potential, New Model of Parkinson’s Disease. Front. Biosci..

[119] Jangula A., Murphy E.J. (2013). Lipopolysaccharide-Induced Blood Brain Barrier Permeability Is Enhanced by Alpha-Synuclein Expression. Neurosci. Lett..

[120] Portincasa P., Bonfrate L., Vacca M., De Angelis M., Farella I., Lanza E., Khalil M., Wang D.Q.H., Sperandio M., Di Ciaula A. (2022). Gut Microbiota and Short Chain Fatty Acids: Implications in Glucose Homeostasis. Int. J. Mol. Sci..

[121] Liu H., Wang J., He T., Becker S., Zhang G., Li D., Ma X. (2018). Butyrate: A Double-Edged Sword for Health?. Adv. Nutr..

[122] Zhang K., Ji X., Song Z., Song W., Huang Q., Yu T., Shi D., Wang F., Xue X., Guo J. (2023). Butyrate Inhibits the Mitochondrial Complex Ι to Mediate Mitochondria-Dependent Apoptosis of Cervical Cancer Cells. BMC Complement. Med. Ther..

[123] Bienenstock J., Kunze W., Forsythe P. (2015). Microbiota and the Gut-Brain Axis. Nutr. Rev..

[124] Lotankar S., Prabhavalkar K.S., Bhatt L.K. (2017). Biomarkers for Parkinson’s Disease: Recent Advancement. Neurosci. Bull..

[125] Le W., Dong J., Li S., Korczyn A.D. (2017). Can Biomarkers Help the Early Diagnosis of Parkinson’s Disease?. Neurosci. Bull..

[126] Tolosa E., Garrido A., Scholz S.W., Poewe W. (2021). Challenges in the Diagnosis of Parkinson’s Disease. Lancet Neurol..

[127] Delenclos M., Jones D.R., McLean P.J., Uitti R.J. (2016). Biomarkers in Parkinson’s Disease: Advances and Strategies. Park. Relat. Disord..

[128] Dos Santos M.C.T., Scheller D., Schulte C., Mesa I.R., Colman P., Bujac S.R., Bell R., Berteau C., Perez L.T., Lachmann I. (2018). Evaluation of Cerebrospinal Fluid Proteins as Potential Biomarkers for Early Stage Parkinson’s Disease Diagnosis. PLoS ONE.

[129] Magalhães P., Lashuel H.A. (2022). Opportunities and Challenges of Alpha-Synuclein as a Potential Biomarker for Parkinson’s Disease and Other Synucleinopathies. npj Park. Dis..

[130] Kwon E.H., Tennagels S., Gold R., Gerwert K., Beyer L., Tönges L. (2022). Update on CSF Biomarkers in Parkinson’s Disease. Biomolecules.

[131] Siderowf A., Concha-Marambio L., Lafontant D.E., Farris C.M., Ma Y., Urenia P.A., Nguyen H., Alcalay R.N., Chahine L.M., Foroud T. (2023). Assessment of Heterogeneity among Participants in the Parkinson’s Progression Markers Initiative Cohort Using α-Synuclein Seed Amplification: A Cross-Sectional Study. Lancet Neurol..

[132] Li Q., Meng L.B., Chen L.J., Shi X., Tu L., Zhou Q., Yu J.L., Liao X., Zeng Y., Yuan Q.Y. (2023). The Role of the Microbiota-Gut-Brain Axis and Intestinal Microbiome Dysregulation in Parkinson’s Disease. Front. Neurol..

[133] Claudino Dos Santos J.C., Oliveira L.F., Noleto F.M., Gusmão C.T.P., de Castro Brito G.A., de Barros Viana G.S. (2023). Gut-Microbiome-Brain Axis: The Crosstalk between the Vagus Nerve, Alpha-Synuclein and the Brain in Parkinson’s Disease. Neural Regen. Res..

[134] Weis S., Schwiertz A., Unger M.M., Becker A., Faßbender K., Ratering S., Kohl M., Schnell S., Schäfer K.-H., Egert M. (2019). Effect of Parkinson’s Disease and Related Medications on the Composition of the Fecal Bacterial Microbiota. npj Park. Dis..

[135] Zhao Y., Yu Y.-B. (2016). Intestinal Microbiota and Chronic Constipation. Springerplus.

[136] Salim S., Ahmad F., Banu A., Mohammad F. (2023). Gut Microbiome and Parkinson’s Disease: Perspective on Pathogenesis and Treatment. J. Adv. Res..

[137] Gorecki A.M., Preskey L., Bakeberg M.C., Kenna J.E., Gildenhuys C., MacDougall G., Dunlop S.A., Mastaglia F.L., Anthony Akkari P., Koengten F. (2019). Altered Gut Microbiome in Parkinson’s Disease and the Influence of Lipopolysaccharide in a Human α-Synuclein over-Expressing Mouse Model. Front. Neurosci..

[138] Yang H., Li S., Le W. (2022). Intestinal Permeability, Dysbiosis, Inflammation and Enteric Glia Cells: The Intestinal Etiology of Parkinson’s Disease. Aging Dis..

[139] Haikal C., Chen Q.-Q., Li J.-Y. (2019). Microbiome Changes: An Indicator of Parkinson’s Disease?. Transl. Neurodegener..

[140] Yan J., Feng X., Zhou X., Zhao M., Xiao H., Li R., Shen H. (2022). Identification of Gut Metabolites Associated with Parkinson’s Disease Using Bioinformatic Analyses. Front. Aging Neurosci..

[141] Qian Y., Yang X., Xu S., Huang P., Li B., Du J., He Y., Su B., Xu L.M., Wang L. (2020). Gut Metagenomics-Derived Genes as Potential Biomarkers of Parkinson’s Disease. Brain.

[142] Hashish S., Salama M. (2023). The Role of an Altered Gut Microbiome in Parkinson’s Disease: A Narrative Review. Appl. Microbiol..

[143] Zacharias H.U., Kaleta C., Cossais F., Schaeffer E., Berndt H., Best L., Dost T., Glüsing S., Groussin M., Poyet M. (2022). Microbiome and Metabolome Insights into the Role of the Gastrointestinal–Brain Axis in Parkinson’s and Alzheimer’s Disease: Unveiling Potential Therapeutic Targets. Metabolites.

[144] Forero-Rodríguez J., Zimmermann J., Taubenheim J., Arias-Rodríguez N., Caicedo-Narvaez J.D., Best L., Mendieta C.V., López-Castiblanco J., Gómez-Muñoz L.A., Gonzalez-Santos J. (2024). Changes in Bacterial Gut Composition in Parkinson’s Disease and Their Metabolic Contribution to Disease Development: A Gut Community Reconstruction Approach. Microorganisms.

[145] Yamashita K.Y., Bhoopatiraju S., Silverglate B.D., Grossberg G.T. (2023). Biomarkers in Parkinson’s Disease: A State of the Art Review. Biomark. Neuropsychiatry.

[146] Jankovic J., Aguilar L.G. (2008). Current Approaches to the Treatment of Parkinson’s Disease. Neuropsychiatr. Dis. Treat..

[147] Poewe W., Antonini A., Zijlmans J.C., Burkhard P.R., Vingerhoets F. (2010). Levodopa in the Treatment of Parkinson’s Disease: An Old Drug Still Going Strong. Clin. Interv. Aging.

[148] Mulak A., Bonaz B. (2015). Brain-Gut-Microbiota Axis in Parkinson’s Disease. World J. Gastroenterol..

[149] Toledo A.R.L., Monroy G.R., Salazar F.E., Lee J.Y., Jain S., Yadav H., Borlongan C.V. (2022). Gut–Brain Axis as a Pathological and Therapeutic Target for Neurodegenerative Disorders. Int. J. Mol. Sci..

[150] Gulliver E.L., Young R.B., Chonwerawong M., D’Adamo G.L., Thomason T., Widdop J.T., Rutten E.L., Rossetto Marcelino V., Bryant R.V., Costello S.P. (2022). Review Article: The Future of Microbiome-Based Therapeutics. Aliment. Pharmacol. Ther..

[151] Aggarwal N., Kitano S., Puah G.R.Y., Kittelmann S., Hwang I.Y., Chang M.W. (2023). Microbiome and Human Health: Current Understanding, Engineering, and Enabling Technologies. Chem. Rev..

[152] Bajaj J.S., Ng S.C., Schnabl B. (2022). Promises of Microbiome-Based Therapies. J. Hepatol..

[153] Wong A.C. New Approaches to Microbiome-Based Therapies ABSTRACT.

[154] Bruggeman A., Vandendriessche C., Hamerlinck H., De Looze D., Tate D.J., Vuylsteke M., De Commer L., Devolder L., Raes J., Verhasselt B. (2024). Safety and Efficacy of Faecal Microbiota Transplantation in Patients with Mild to Moderate Parkinson’s Disease (GUT-PARFECT): A Double-Blind, Placebo-Controlled, Randomised, Phase 2 Trial. eClinicalMedicine.

[155] Jackson A., Forsyth C.B., Shaikh M., Voigt R.M., Engen P.A., Ramirez V., Keshavarzian A. (2019). Diet in Parkinson’s Disease: Critical Role for the Microbiome. Front. Neurol..

[156] Hegelmaier T., Lebbing M., Duscha A., Tomaske L., Tönges L., Holm J.B., Bjørn Nielsen H., Gatermann S.G., Przuntek H., Haghikia A. (2020). Interventional Influence of the Intestinal Microbiome Through Dietary Intervention and Bowel Cleansing Might Improve Motor Symptoms in Parkinson’s Disease. Cells.

[157] Singh R.K., Chang H.W., Yan D., Lee K.M., Ucmak D., Wong K., Abrouk M., Farahnik B., Nakamura M., Zhu T.H. (2017). Influence of Diet on the Gut Microbiome and Implications for Human Health. J. Transl. Med..

[158] Purdel C., Margină D., Adam-Dima I., Ungurianu A. (2023). The Beneficial Effects of Dietary Interventions on Gut Microbiota—An Up-to-Date Critical Review and Future Perspectives. Nutrients.

[159] Leeming E.R., Johnson A.J., Spector T.D., Roy C.I.L. (2019). Effect of Diet on the Gut Microbiota: Rethinking Intervention Duration. Nutrients.

[160] Duan W.X., Wang F., Liu J.Y., Liu C.F. (2023). Relationship Between Short-Chain Fatty Acids and Parkinson’s Disease: A Review from Pathology to Clinic. Neurosci. Bull..

[161] Suganya K., Koo B.S. (2020). Gut–Brain Axis: Role of Gut Microbiota on Neurological Disorders and How Probiotics/Prebiotics Beneficially Modulate Microbial and Immune Pathways to Improve Brain Functions. Int. J. Mol. Sci..

[162] Koh A., De Vadder F., Kovatcheva-Datchary P., Bäckhed F. (2016). From Dietary Fiber to Host Physiology: Short-Chain Fatty Acids as Key Bacterial Metabolites. Cell.

[163] Makki K., Deehan E.C., Walter J., Bäckhed F. (2018). The Impact of Dietary Fiber on Gut Microbiota in Host Health and Disease. Cell Host Microbe.

[164] Perez-Pardo P., de Jong E.M., Broersen L.M., van Wijk N., Attali A., Garssen J., Kraneveld A.D. (2017). Promising Effects of Neurorestorative Diets on Motor, Cognitive, and Gastrointestinal Dysfunction after Symptom Development in a Mouse Model of Parkinson’s Disease. Front. Aging Neurosci..

[165] Wang Q., Luo Y., Ray Chaudhuri K., Reynolds R., Tan E.K., Pettersson S. (2021). The Role of Gut Dysbiosis in Parkinson’s Disease: Mechanistic Insights and Therapeutic Options. Brain.

[166] Socała K., Doboszewska U., Szopa A., Serefko A., Włodarczyk M., Zielińska A., Poleszak E., Fichna J., Wlaź P. (2021). The Role of Microbiota-Gut-Brain Axis in Neuropsychiatric and Neurological Disorders. Pharmacol. Res..

[167] Soundharrajan I., Kuppusamy P., Srisesharam S., Lee J.C., Sivanesan R., Kim D., Choi K.C. (2020). Positive Metabolic Effects of Selected Probiotic Bacteria on Diet-Induced Obesity in Mice Are Associated with Improvement of Dysbiotic Gut Microbiota. FASEB J..

[168] Alard J., Lehrter V., Rhimi M., Mangin I., Peucelle V., Abraham A.L., Mariadassou M., Maguin E., Waligora-Dupriet A.J., Pot B. (2016). Beneficial Metabolic Effects of Selected Probiotics on Diet-Induced Obesity and Insulin Resistance in Mice Are Associated with Improvement of Dysbiotic Gut Microbiota. Environ. Microbiol..

[169] Lombardi F., Fiasca F., Minelli M., Maio D., Mattei A., Vergallo I., Cifone M.G., Cinque B., Minelli M. (2020). The Effects of Low-Nickel Diet Combined with Oral Administration of Selected Probiotics on Patients with Systemic Nickel Allergy Syndrome (SNAS) and Gut Dysbiosis. Nutrients.

[170] Lubomski M., Davis R.L., Sue C.M. (2019). The Gut Microbiota: A Novel Therapeutic Target in Parkinson’s Disease?. Park. Relat. Disord..

[171] Rekdal V.M., Bess E.N., Bisanz J.E., Turnbaugh P.J., Balskus E.P. (2019). Discovery and Inhibition of an Interspecies Gut Bacterial Pathway for Levodopa Metabolism. Science.

[172] van Kessel S.P., Frye A.K., El-Gendy A.O., Castejon M., Keshavarzian A., van Dijk G., El Aidy S. (2019). Gut Bacterial Tyrosine Decarboxylases Restrict Levels of Levodopa in the Treatment of Parkinson’s Disease. Nat. Commun..

[173] Liu H., Su W., Li S., Du W., Ma X., Jin Y., Li K., Chen H. (2017). Eradication of Helicobacter Pylori Infection Might Improve Clinical Status of Patients with Parkinson’s Disease, Especially on Bradykinesia. Clin. Neurol. Neurosurg..

[174] Lolekha P., Sriphanom T., Vilaichone R.K. (2021). Helicobacter Pylori Eradication Improves Motor Fluctuations in Advanced Parkinson’s Disease Patients: A Prospective Cohort Study (HP-PD Trial). PLoS ONE.

[175] Hashim H., Azmin S., Razlan H., Yahya N.W., Tan H.J., Manaf M.R.A., Ibrahim N.M. (2014). Eradication of Helicobacter Pylori Infection Improves Levodopa Action, Clinical Symptoms and Quality of Life in Patients with Parkinson’s Disease. PLoS ONE.

[176] Fasano A., Visanji N.P., Liu L.W.C., Lang A.E., Pfeiffer R.F. (2015). Gastrointestinal Dysfunction in Parkinson’s Disease. Lancet Neurol..

[177] Boelens Keun J.T., Arnoldussen I.A., Vriend C., Van De Rest O. (2021). Dietary Approaches to Improve Efficacy and Control Side Effects of Levodopa Therapy in Parkinson’s Disease: A Systematic Review. Adv. Nutr..

[178] Vaswani P.A., Tropea T.F., Dahodwala N. (2020). Overcoming Barriers to Parkinson Disease Trial Participation: Increasing Diversity and Novel Designs for Recruitment and Retention. Neurotherapeutics.

[179] Zhang F., Yue L., Fang X., Wang G., Li C., Sun X., Jia X., Yang J., Song J., Zhang Y. (2020). Altered Gut Microbiota in Parkinson’s Disease Patients/Healthy Spouses and Its Association with Clinical Features. Park. Relat. Disord..

[180] Fonseca D.C., Marques Gomes da Rocha I., Depieri Balmant B., Callado L., Aguiar Prudêncio A.P., Tepedino Martins Alves J., Torrinhas R.S., da Rocha Fernandes G., Linetzky Waitzberg D. (2024). Evaluation of Gut Microbiota Predictive Potential Associated with Phenotypic Characteristics to Identify Multifactorial Diseases. Gut Microbes.

[181] Mathur S., Sutton J. (2017). Personalized Medicine Could Transform Healthcare (Review). Biomed. Reports.

[182] Naylor S., Chen J.Y. (2010). Unraveling Human Complexity and Disease with Systems Biology and Personalized Medicine. Per. Med..

[183] Goetz L.H., Schork N.J. (2018). Personalized Medicine: Motivation, Challenges, and Progress. Fertil. Steril..

[184] Zhao Q., Chen Y., Huang W., Zhou H., Zhang W. (2023). Drug-Microbiota Interactions: An Emerging Priority for Precision Medicine. Signal Transduct. Target. Ther..

[185] Wilson I.D., Nicholson J.K. (2017). Gut Microbiome Interactions with Drug Metabolism, Efficacy and Toxicity Europe PMC Funders Author Manuscripts The Gut Microbiota Have the Capability of Preforming a Wide Range of Metabolic Reactions On. Transl. Res..

[186] Walsh J., Griffin B.T., Clarke G., Hyland N.P. (2018). Drug–Gut Microbiota Interactions: Implications for Neuropharmacology. Br. J. Pharmacol..

[187] Wan Y., Zuo T. (2022). Interplays between Drugs and the Gut Microbiome. Gastroenterol. Rep..

[188] Dutta D., Lim S.H. (2020). Bidirectional Interaction between Intestinal Microbiome and Cancer: Opportunities for Therapeutic Interventions. Biomark. Res..

[189] Weersma R.K., Zhernakova A., Fu J. (2020). Interaction between Drugs and the Gut Microbiome. Gut.

[190] Misera A., Łoniewski I., Palma J., Kulaszyńska M., Czarnecka W., Kaczmarczyk M., Liśkiewicz P., Samochowiec J., Skonieczna-Żydecka K. (2023). Clinical Significance of Microbiota Changes under the Influence of Psychotropic Drugs. An Updated Narrative Review. Front. Microbiol..

[191] Enright E.F., Gahan C.G.M., Joyce S.A., Griffin B.T. (2016). The Impact of the Gut Microbiota on Drug Metabolism and Clinical Outcome. Yale, J. Biol. Med..

[192] Fan H.X., Sheng S., Zhang F. (2022). New Hope for Parkinson’s Disease Treatment: Targeting Gut Microbiota. CNS Neurosci. Ther..

[193] Shen T., Yue Y., He T., Huang C., Qu B., Lv W., Lai H.Y. (2021). The Association Between the Gut Microbiota and Parkinson’s Disease, a Meta-Analysis. Front. Aging Neurosci..

[194] Keshavarzian A., Green S.J., Engen P.A., Voigt R.M., Naqib A., Forsyth C.B., Mutlu E., Shannon K.M. (2015). Colonic Bacterial Composition in Parkinson’s Disease. Mov. Disord..

[195] Huang H., Jiang J., Wang X., Jiang K., Cao H. (2024). Exposure to Prescribed Medication in Early Life and Impacts on Gut Microbiota and Disease Development. eClinicalMedicine.

[196] WADE D.N., MEARRICK P.T., MORRIS J.L. (1973). Active Transport of L-Dopa in the Intestine. Nature.

[197] Ngwuluka N., Pillay V., Du Toit L.C., Ndesendo V., Choonara Y., Modi G., Naidoo D. (2010). Levodopa Delivery Systems: Advancements in Delivery of the Gold Standard. Expert Opin. Drug Deliv..

[198] Smith-Hicks C., Raymond G.V., Irani D. (2009). Developmental Disorders. Cerebrospinal Fluid in Clinical Practice.

[199] Gonçalves S., Nunes-Costa D., Cardoso S.M., Empadinhas N., Marugg J.D. (2022). Enzyme Promiscuity in Serotonin Biosynthesis, From Bacteria to Plants and Humans. Front. Microbiol..

[200] Gershanik O.S. (2015). Improving L-Dopa Therapy: The Development of Enzyme Inhibitors. Mov. Disord..

[201] Jenner P. (2023). Stalevo(®): A Pioneering Treatment for OFF Periods in Parkinsons Disease. Eur. J. Neurol..

[202] Tambasco N., Romoli M., Calabresi P. (2017). Levodopa in Parkinson’s Disease: Current Status and Future Developments. Curr. Neuropharmacol..

[203] Tsunoda S.M., Gonzales C., Jarmusch A.K., Momper J.D., Ma J.D. (2021). Contribution of the Gut Microbiome to Drug Disposition, Pharmacokinetic and Pharmacodynamic Variability. Clin. Pharmacokinet..

[204] Xu K., Sheng S., Zhang F. (2022). Relationship Between Gut Bacteria and Levodopa Metabolism. Curr. Neuropharmacol..

[205] Jameson K.G., Hsiao E.Y. (2019). A Novel Pathway for Microbial Metabolism of Levodopa. Nat. Med..

[206] Zimmermann M., Zimmermann-Kogadeeva M., Wegmann R., Goodman A.L. (2019). Mapping Human Microbiome Drug Metabolism by Gut Bacteria and Their Genes. Nature.

